# Urothelial collective-gliding response acts as a toll-like receptor 4-associated defense mechanism

**DOI:** 10.1016/j.isci.2025.113553

**Published:** 2025-09-12

**Authors:** Ning Zhang, Takeshi Sano, Katsuhiro Ito, Shinji Ito, Ryosuke Ikeuchi, Hideaki Takada, Kenji Nakamura, Toru Sakatani, Akihiro Hamada, Masashi Takeda, Kaoru Murakami, Yuki Kita, Takayuki Sumiyoshi, Takayuki Goto, Ryoichi Saito, Osamu Ogawa, Michiyuki Matsuda, Takashi Kobayashi

**Affiliations:** 1 Department of Urology, Graduate School of Medicine, Kyoto University, Kyoto, Japan; 2 Department of Pathology and Biology of Diseases, Graduate School of Medicine, Kyoto University, Kyoto, Japan; 3 Department of Urology and Andrology, Kansai Medical University, Osaka, Japan; 4 Department of Immunology and Genome Medicine, Graduate School of Medicine, Kyoto University, Kyoto, Japan; 5 Medical Research Support Center, Graduate School of Medicine, Kyoto University, Kyoto, Japan

**Keywords:** Biochemistry, Cell biology

## Abstract

Collective cell migration (CCM) is characterized by the coordinated movement of cell groups while maintaining cell-to-cell cohesion. Despite extensive research on CCM, the collective migration of mature epithelial cells over the extracellular matrix in response to external stimuli has not been reported. Using intravital imaging in mice, we identified urothelial CCM (UCCM) triggered by immunogenic substances, including bladder cancer cells (MB49) and uropathogenic *Escherichia coli* (UPEC). Integrin signaling inhibitors suppress UCCM, significantly enhancing MB49 tumor growth and UPEC bladder infection. UCCM initiation involves Toll-like receptor 4 (TLR4), we designated this TLR4-associated UCCM as the urothelial collective-gliding response (UCGR). Downstream of integrin signaling, urothelial matrix metalloproteinases (MMP)-8 and MMP-9 mediate UCGR. Intravesical instillation of these factors accelerates UCCM and inhibits tumor growth and infection. UCGR may represent a TLR4-associated defense mechanism, offering potential therapeutic strategies for bladder disorders such as refractory cystitis and recurrent non-muscle invasive bladder cancer after endoscopic resection.

## Highlights

• Exposure to immunogenic substances induces urothelial collective cell migration (UCCM)
• Immunogen-induced UCCM was termed the urothelial collective-gliding response (UCGR)
• UCGR is a defense mechanism associated with the innate immune response
• UCCM suppresses tumorigenesis and infection through mechanisms that remain to be elucidated

## Graphical abstract

**Figure:**
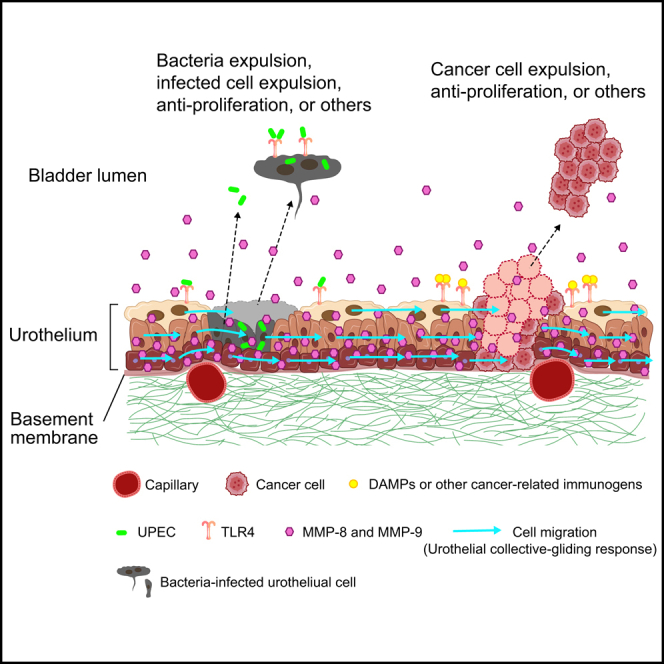

## Introduction

Collective cell migration (CCM) refers to the coordinated movement of a group of cells while maintaining functional cell-to-cell cohesion.^1^^,^^2^^,^^3^ While the fundamental steps of single-cell migration, such as formation of cell polarization, protrusion, and the attachment of the leading edge to the substrate via integrin-based mechanosensory interaction, and actomyosin contraction for retraction of cell rear, are preserved,^4^ cadherin-mediated cell-cell adhesion complexes and the differential localization of Rho GTPases within cells facilitate the coordination of cell polarization and directional migration in groups.^5^ Several external cues, including topographic information sensed by adhesion receptors and gradients of chemo-attractants, guide the coordinated migration of cell groups.^5^ Integrin-associated focal adhesion proteins, such as focal adhesion kinase (FAK), Src, and ERK/MAPK, regulate cell-extracellular matrix (ECM) adhesion, a critical process for cell migration.^6^ CCM plays a vital role in physiological and pathological processes, including embryonic development, epithelial wound healing, and cancer cell invasion.^1^^,^^2^^,^^3^ During epidermal wound healing, a multilayered epithelium migrates directionally over the ECM, which is synthesized to close the wound gap.^7^

Mucosal immunity in the bladder comprises multiple defense components, including antimicrobial peptides, secretory immunoglobulins, and immune cells.^8^ Upon exposure to immunogenic substances, pattern recognition receptors, particularly Toll-like receptors (TLRs), initiate an innate immune response. Although TLRs are expressed in both immune and urothelial cells, the functional role of their urothelial expression remains largely unclear. Among these receptors, TLR4, which binds to LPS and detects uropathogenic *Escherichia coli* (UPEC), is the most extensively studied.^9^^,^^10^ Upon recognizing immunogenic substances, urothelial TLR4 activates a cascade of defense responses, including the secretion of antimicrobial peptides and various cytokines and chemokines. These signaling molecules mediate cell-cell communication during immune responses and promote the proliferation and recruitment of innate immune cells, thereby bolstering the antimicrobial defense system of the bladder.

The mouse urothelium exhibits a three-layered structure consisting of umbrella, intermediate, and basal cells. Basal cells attach to the basement membrane via focal adhesions, whereas some intermediate cells attach to the basement membrane through slender projections,^11^ collectively ensuring robust urothelial attachment. To investigate urothelial dynamics, we established an intravital imaging method using two-photon excitation microscopy (TPEM), which is particularly well-suited for studying mammalian cell migration across various organs.^12^^,^^13^ Using this approach, we identified a unique mode of urothelial CCM (UCCM) during urothelial wound healing.^14^^,^^15^ In this study, we demonstrate that various immunogenic substances can trigger UCCM in a TLR4-dependent manner, and inhibiting UCCM exacerbates tumor growth and UPEC infection, suggesting its potential role as a novel defense mechanism in the bladder. Notably, to our knowledge, CCM has not been previously reported as a defensive mechanism in any epithelial tissue. This study aims to elucidate the mechanisms underlying pathogen-induced UCCM and its biological significance.

## Results

### Urothelial cells exhibit collective migration over ECM in tumor-bearing mouse bladders

**Figure 1:**
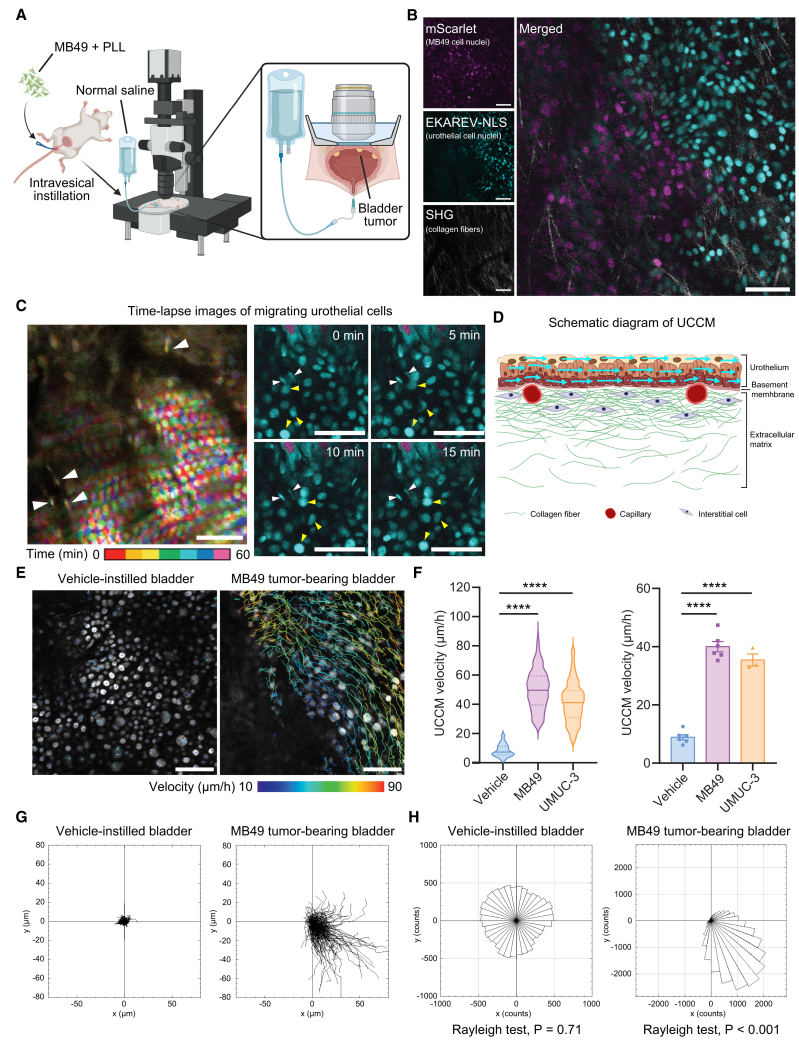
Urothelial cells exhibit collective migration over ECM in tumor-bearing mouse bladders (A) Schematic of the intravital imaging system for the orthotopic bladder cancer mouse model (created with BioRender.com). (B) Representative TPEM image showing both MB49 tumor region and adjacent normal urothelium in the basal cell layer immediately above the ECM. MB49 cell nuclei are shown in magenta (mScarlet) and normal urothelial cell nuclei in cyan (EKAREV-NLS) and fibrillar collagens in the ECM in white (SHG). (C–H) Comparative analysis of urothelial cell dynamics: in tumor-bearing bladders, urothelial cells displayed collective migration over the ECM, whereas cells in normal bladders remained stationary. (C) Left: Composite image showing 24 time-series frames at the urothelium-ECM interface, with seven time-coded colors as indicated by the color bar. Stationary endothelial cells appear white due to their non-migratory elongated nuclei (white arrowheads). Right: representative time-lapse images depicting MB49 cells (magenta), endothelial cells (white arrowheads, cyan), and migrating urothelial cells (yellow arrowheads, cyan). (D) Schematic representation of UCCM. (E) Representative urothelial tracking images in vehicle-instilled and MB49 tumor-bearing bladders 3 days after intravesical instillation. Migration trajectories of urothelial cells during 2-h intravital imaging are shown as color-coded lines, with colors indicating migration velocities. (F) UCCM velocity analysis: (Left) violin plots of individual urothelial cell velocities from representative experiments, with median (solid lines) and IQR (dotted lines). *n* > 250 cells/group. (Right) mean UCCM velocity of all cells per experiment (mean ± SEM). *n* = 6 mice for the vehicle and MB49 groups, and *n* = 3 mice for UMUC-3 group. (G and H) Cell trajectory analysis showing nuclear centroid displacement during 2-h imaging (G) and rose diagrams illustrating directional preferences of urothelial cell migration (H) in representative experiments. Scale bars: 100 μm. One-way ANOVA with Dunnett’s multiple comparisons test (F) and Rayleigh test (H). ∗∗∗∗*p* < 0.0001.

We employed a modified orthotopic bladder cancer mouse model using the MB49 cell line,^16^ a murine bladder cancer cell line that reliably develops bladder tumors approximately 3 days following intravesical pretreatment with poly-L-lysine (PLL) and subsequent intravesical MB49 instillation (Figure 1A). Using our previously established intravital imaging protocol for the mouse bladder,^15^ we visualized MB49 cell nuclei labeled with the red fluorescent protein mScarlet, urothelial cell nuclei labeled with the Förster resonance energy transfer (FRET) biosensor for ERK activity (EKAREV-NLS),^17^ and fibrillar collagens with second-harmonic generation (SHG) fluorescence (Figure 1B).

Time-lapse imaging over a 2-h period on the 3rd day post-MB49 instillation revealed that urothelial cells near MB49 tumor cells collectively migrated over the suburothelial capillary plexus and fibrillar collagens in the ECM (Figures 1C and 1D; Video S1). Time-lapse images of a single plane captured both urothelial and endothelial cells of the subepithelial capillaries. Although urothelial cells collectively migrate, endothelial cells remain stationary, confirming that urothelial cells migrated over the ECM during UCCM (Figure 1C). Almost all urothelial cells within the imaged were in motion, and adjacent cells migrated in similar directions (Figures 1E and 1F). The average velocity of UCCM across six imaging sessions was approximately 40 μm/h (Figure 1F). In contrast, urothelial cells in vehicle-instilled mice did not exhibit CCM (Figures 1E and 1F; Video S2). Notably, UCCM occurred independently of the proximity to MB49 tumors (Figure S1A) and was also observed in mouse bladders bearing UMUC-3, a human bladder cancer cell line (Figure 1F). Trajectory tracking of migrating cells confirmed the directional migrations of urothelial cells in tumor-bearing mice (Figures 1E–1G, and 1H; Figure S1B and S1C). Although we previously observed sheet-like gliding migration of urothelial cells over ECM during wound healing,^14^ UCCM associated with bladder tumor development has not been reported. Thus, we further investigated the mechanisms driving UCCM and its clinical implications in tumor-bearing mice.

Video S1. Time-lapse video of intravital imaging showing UCCM in MB49 tumor-bearing mouse bladder, relating to Figure 1 The boundary between clustered MB49 cells is shown in magenta. In contrast, normal urothelial cells are shown in cyan at the basal cell layer in the mouse bladder 3 days after intravesical MB49 instillation with PLL pretreatment. Time in h/min. Scale bar: 100 μm. The playback speed was set to 12 frames per sec.

Video S2. Time-lapse video of intravital imaging showing urothelial cells in the control mouse bladder, relating to Figure 1 Urothelial cells are shown in cyan at the basal cell layer in the mouse bladder 3 days after intravesical PLL treatment alone. Time in h/min. Scale bar: 100 μm. The playback speed was set to 12 frames per sec.

### UCCM primarily depends on the FAK/Src pathway and partially on the ERK pathway

**Figure 2:**
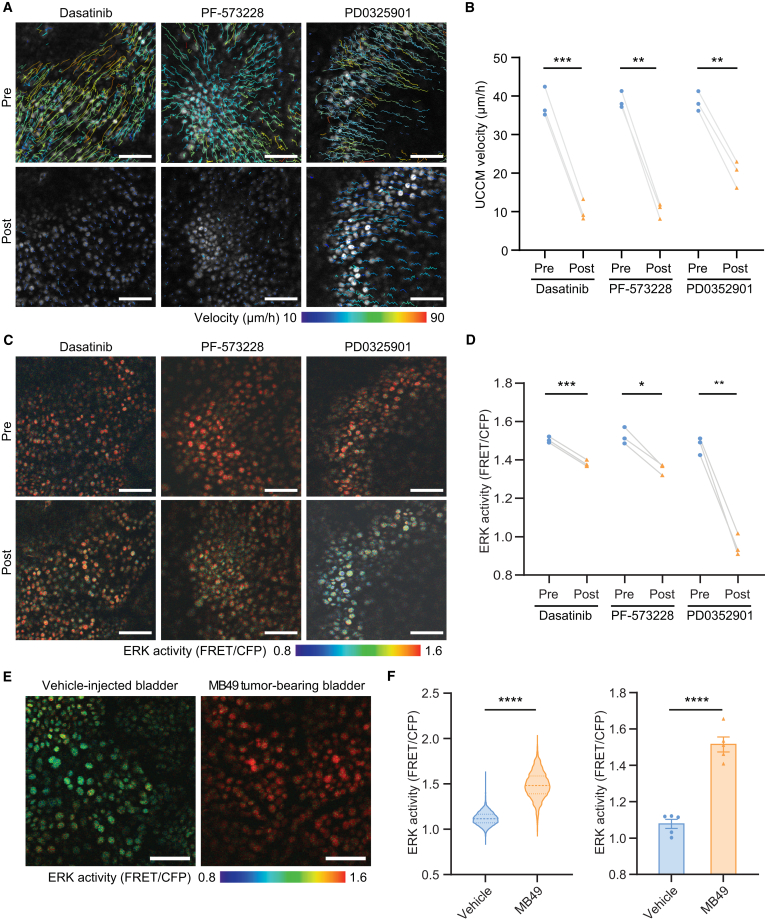
UCCM primarily depends on the FAK/Src pathway and partially on the ERK pathway (A–D) Analysis of UCCM response to i.v. administration of specific inhibitors dasatinib (Src inhibitor), PF-573228 (FAK inhibitor), and PD0325901 (MEK inhibitor). (A) Representative urothelial tracking images in MB49 tumor-bearing bladders before and after inhibitor administration. Cell migration trajectories during 2-h intravital imaging are depicted as color-coded lines, with colors indicating migration velocities. (B) Quantification of mean UCCM velocity in MB49 tumor-bearing bladders pre- and post-inhibitor administration across 2-h intravital imaging periods. *n* = 3 mice/group. (C) Representative FRET/CFP ratio images of the urothelium from the same experiments shown in A, obtained pre- and 1 h post-inhibitor administration. ERK activity is visualized through the pseudo-color representation of individual cells. (D) Quantification of mean FRET/CFP ratio pre and 1 h post inhibitor administration in MB49 tumor-bearing bladders. *n* = 3 mice/group. (E) Representative FRET/CFP ratio images of urothelium at 3 days post-treatment, comparing vehicle vs. PLL and MB49. (F) ERK activity analysis: (Left) violin plots of individual cell ERK activity from a single representative 2-h imaging per group. Data are presented as median (solid lines) and IQR (dotted lines). *n* > 1500 cells/group. (Right) mean ERK activity of all cells per 2-h imaging. Data are presented as mean ± SEM. *n* = 5 mice/group. Scale bars: 100 μm. Paired two-sided student’s t test (B and D) and unpaired two-sided student’s t test (F). ∗*p* < 0.05, ∗∗*p* < 0.01, ∗∗∗*p* < 0.001, ∗∗∗∗*p* < 0.0001.

Integrin signaling is a critical driver of cell migration.^18^ Tyrosine kinases such as FAK and Src regulate CCM through integrin-ECM interactions^19^^,^^20^ and actomyosin contractility.^21^ The ERK MAPK pathway is activated during directional CCM.^22^^,^^23^^,^^24^ To determine the molecular mechanisms underlying UCCM in bladder tumor-bearing mice, we evaluated the effect of FAK and Src inhibitors on UCCM. Intravenous (i.v.) administration of either the Src inhibitor dasatinib or the FAK inhibitor PF-573228 markedly reduced UCCM velocity by >70% (Figures 2A and 2B; Video S3) and mildly decreased ERK activity (Figures 2C and 2D).

Video S3. Time-lapse video of intravital imaging showing UCCM in MB49 tumor-bearing mouse bladder, relating to Figure 2A The video was captured before and after intravenous administration of dasatinib, 3 days after intravesical MB49 instillation with PLL pretreatment. Urothelial cells are shown in cyan, and Qtracker 655, co-administered with dasatinib, is shown in magenta. Time in h/min. Scale bar: 100 μm. The playback speed was set to 12 frames per sec.

Notably, our previous findings demonstrated that CCM in wounded mouse skin requires ERK activation waves propagated from the wound edge.^22^ In contrast, UCCM in the wounded bladder did not exhibit ERK activation waves, and ERK suppression on UCCM had a less pronounced effect compared to its role in skin epithelial cells.^14^ In bladder tumor-bearing mice, ERK activity was elevated in urothelial cells compared to vehicle-instilled mice (Figures 2E and 2F). However, ERK propagation waves were absent (Figures S2A and S2B; Video S4). Intravenous (i.v.) administration of the MAPK/ERK kinase (MEK) inhibitor PD0325901 significantly reduced ERK activity in the urothelium (Figures 2C and 2D). It decreased UCCM velocity by approximately 50% (Figures 2A and 2B). These findings indicate the partial requirement of ERK activity for UCCM.

Video S4. Time-lapse video of intravital imaging showing ERK activity in the urothelium during UCCM in MB49 tumor-bearing bladder, relating to Figure S2 Imaging, was performed 3 days after intravesical MB49 instillation with PLL pre-treatment ERK activity was visualized using a pseudocolor representation according to the FRET/CFP ratio. Warm and cold colors indicate high and low FRET levels, respectively. Time in h/min. Scale bar: 100 μm. The playback speed was set to 12 frames per sec.

### UCCM exerts anti-tumor effects

**Figure 3:**
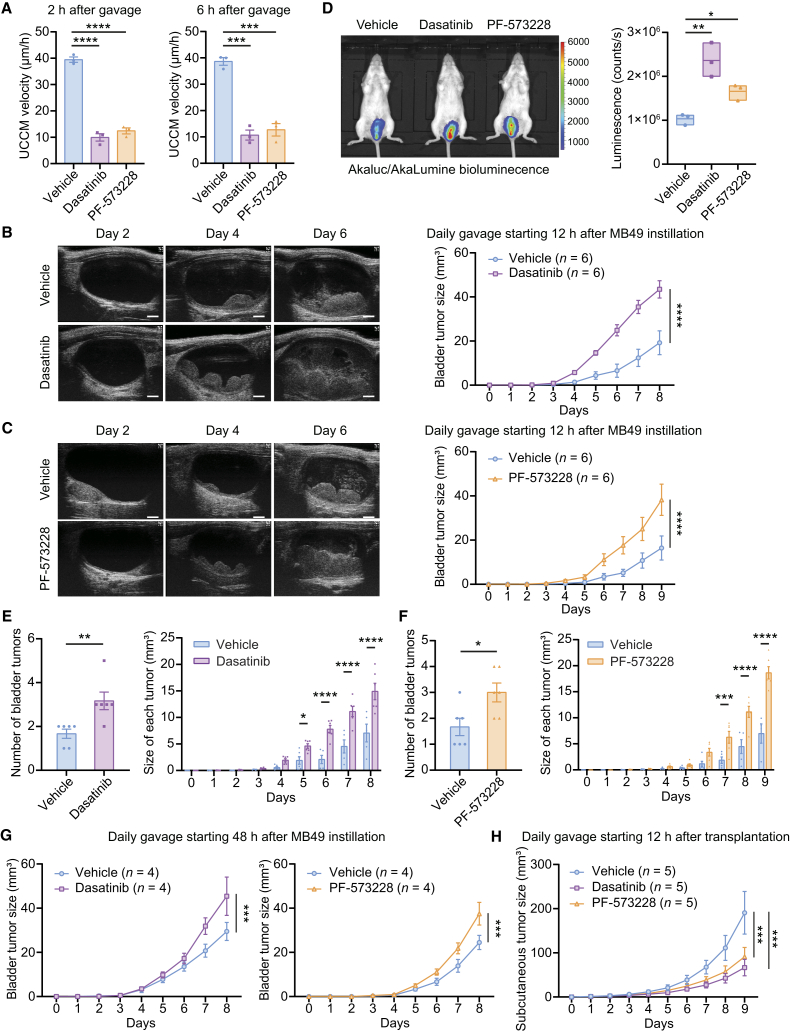
UCCM exerts anti-tumor effects (A) Quantification of mean UCCM velocity in MB49 tumor-bearing bladders at 2 and 6 h post-oral gavage with vehicle, dasatinib (Src inhibitor), or PF-573228 (FAK inhibitor). *n* = 3 mice/group. (B–D) Impact of UCCM inhibition by dasatinib and PF-573228 on MB49 tumor growth. (B and C) Representative ultrasound images of mouse bladders post intravesical instillation of MB49 with PLL pretreatment and quantification of MB49 tumor volumes. Daily gavage of either vehicle or dasatinib (B) and vehicle or PF-573228 (C) commenced 12 h post-MB49 instillation. *n* = 6 mice/group. Scale bars: 1 mm. (D) Representative Akaluc/AkaLumine bioluminescence images and quantification of luminescence counts 9 days post intravesical instillation of MB49 with PLL pretreatment. Daily gavage treatments included vehicle, dasatinib, or PF-573228. The lines of the floating bars indicate the minimum, mean, and maximum values. *n* = 3 mice/group. (E and F) Quantification of the number of bladder tumors in each mouse and the size of each tumor, using the same mice analyzed in B and C. The number of bladder tumors was evaluated with ultrasound imaging 4 days post intravesical instillation of MB49 with PLL pretreatment. Vehicle vs. dasatinib (E) and vehicle vs. PF-573228 (F). (G) Quantification of tumor volumes in mouse bladders post intravesical instillation of MB49 with PLL pretreatment. Daily gavage of either vehicle, dasatinib, or PF-573228 was started 48 h post-MB49 instillation. *n* = 4 mice/group. (H) Quantification of subcutaneous tumor volumes treated with daily gavage of either vehicle, dasatinib, or PF-573228 initiated 12 h post-MB49 transplantation. *n* = 5 mice/group. Data are presented as mean ± SEM. One-way ANOVA with Dunnett’s multiple comparisons test (A and D), two-way ANOVA with Šidák’s test (B, C, and G), unpaired two-sided student’s t test (E and F), and two-way ANOVA with Dunnett’s test (H). ∗*p* < 0.05, ∗∗*p* < 0.01, ∗∗∗*p* < 0.001, ∗∗∗∗*p* < 0.0001.

We investigated the physiological role of UCCM in tumor development. Encouraged by the inhibitory effects of i.v. administration of dasatinib or PF-573228 on UCCM velocity, we administered these compounds via daily oral gavage using a stainless-steel feeding tube. As expected, UCCM velocity in MB49 bladder tumor-bearing mice decreased by over 70% 2 h post gavage with dasatinib or PF-573228 (Figure 3A). This inhibitory effect persisted, with reduced of 72% and 67% observed 6 h post-gavage with dasatinib and PF-573228, respectively (Figure 3A). In randomized, blinded trials, in which researchers performing intravital imaging and data analysis were unaware of treatment groups, daily gavage of dasatinib or PF-573228 led to a more than 2-fold increase in MB49 tumor growth (Figures 3B and 3C). Bioluminescence imaging corroborated these findings, confirming that dasatinib and PF-573228 promoted MB49 tumor growth (Figure 3D). Interestingly, daily gavage of dasatinib or PF-573228 led to an increase in both the number of tumors and the size of each tumor (Figures 3E and 3F). These results were unexpected, given that dasatinib is approved for chronic myeloid lymphoma treatment and has shown potential for other cancers.^25^ FAK inhibitors have demonstrated therapeutic potential in certain solid tumors.^26^ Notably, *in vitro* analysis revealed that dasatinib and PF-573228 exhibited modest anti-proliferative effects on MB49 cells compared to vehicle treatment (Figure S3).

In our protocol, PLL pretreatment before intravesical MB49 instillation disrupted the urothelium and facilitated MB49 engraftment in the bladder. PLL-induced damage has been reported to recover within 6 h.^27^ Since we initiated dasatinib and PF-573228 administration 12 h after intravesical MB49 instillation, it is unlikely that UCCM inhibition delayed urothelial recovery, thereby enhancing tumor growth. Additional experiments, where drug administration commenced 48 h after PLL pretreatment and intravesical MB49 instillation, demonstrated enhanced MB49 tumor growth (Figure 3G). To determine whether UCCM suppression specifically contributed to tumor growth enhancement rather than other inhibitor effects, we evaluated these compounds in a subcutaneous MB49 bladder cancer model. In this context, daily gavage of dasatinib or PF-573228 reduced flank tumor growth compared to vehicle treatment (Figure 3H). Collectively, these results suggest that UCCM plays a defensive role by inhibiting bladder tumor growth.

### UCCM is associated with innate immune response and inflammatory pathways

**Figure 4:**
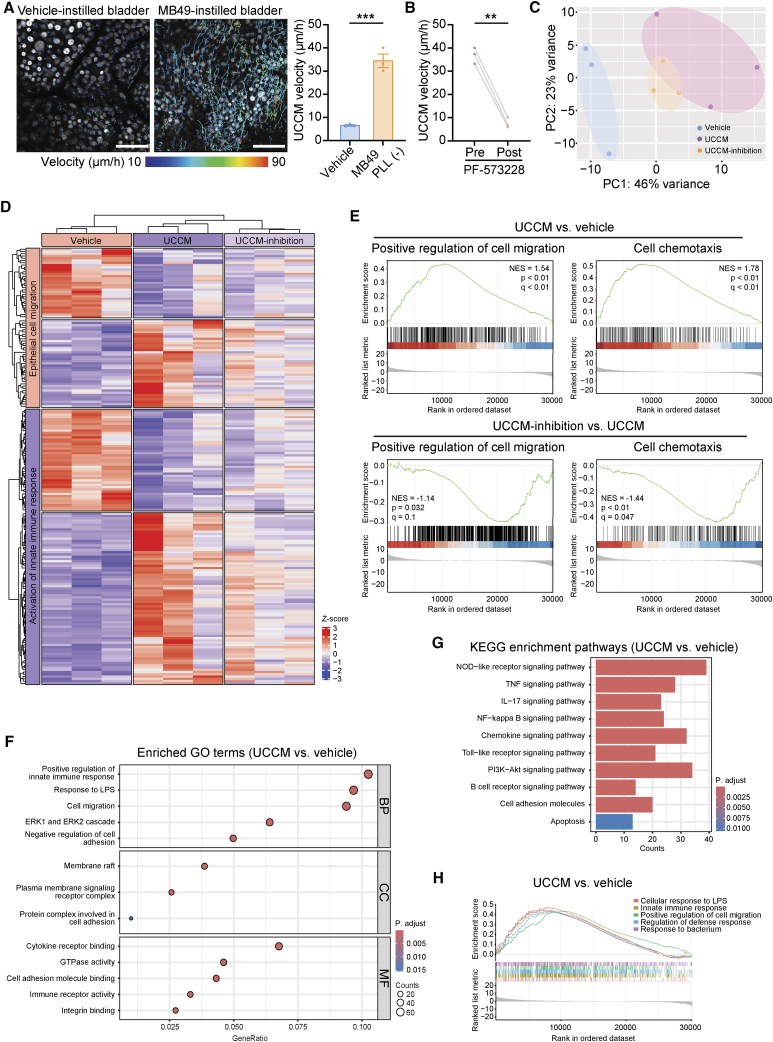
UCCM is associated with innate immune response and inflammatory pathways (A–B) UCCM induced by direct contact between MB49 cells and the urothelium. (A) Representative urothelial tracking images and mean UCCM velocity comparing vehicle-instilled and MB49-instilled bladders without PLL pretreatment. Cell migration trajectories of urothelial cells during 2-h intravital imaging are shown as color-coded lines representing velocities. Data are presented as the mean ± SEM. *n* = 3 mice/group. (B) Effect of intravenous administration of PF-573228 (FAK inhibitor) on the velocity of UCCM induced by intravesical MB49 instillation without PLL pretreatment. Intravital imaging started 4 h after MB49 instillation. Quantification of mean UCCM velocity in MB49-instilled bladders by 2-h intravital imaging pre and post-intravenous administration of PF-573228 in each experiment. *n* = 3 mice/group. Scale bars: 100 μm. Unpaired two-sided Student’s t test (A). Paired two-sided Student’s t test (B). ∗∗*p* < 0.01, ∗∗∗*p* < 0.001. (C–H) Bulk RNA-Seq analysis of urothelium from three experimental groups: vehicle instillation (Vehicle), MB49 instillation (UCCM), MB49 instillation with i.v. PF-573228 (UCCM-inhibition). *n* = 3 mice/group. (C) Principal component analysis of all analyzed genes. (D) Heatmap showing expression profiles for “epithelial cell migration” and “activation of innate immune response” gene sets in the vehicle, UCCM, and UCCM-inhibition groups. (E) GSEA diagrams for “positive regulation of cell migration” and “chemotaxis” gene sets in the UCCM group vs. the vehicle group and the UCCM-inhibition group vs. the UCCM group. (F) GO enrichment analysis of upregulated genes in the UCCM group compared to the vehicle group shown with biological process (BP), cellular component (CC), and molecular function (MF) terms. The colors indicate the statistical significance of each term. The circle size indicates the number of DEGs. (G) KEGG pathway enrichment analysis of upregulated genes in the UCCM group compared to the vehicle group. Only pathways with an adjusted *p* value of <0.05 are shown. Colors indicate the statistical significance of each pathway. (H) GSEA diagram showing enriched gene sets in the UCCM group vs. the vehicle group.

To investigate how urothelial cells respond to MB49 cells and induce integrin signaling-dependent UCCM, we performed bulk RNA sequencing (RNA-seq) on vehicle and MB49-treated urothelia. Before initiating RNA-seq analysis, we addressed potential concerns about RNA contamination from MB49 cells in the treated urothelium. First, we confirmed whether the MB49 application could induce UCCM independently of tumor development. UCCM was observed approximately 4 h after intravesical MB49 instillation without PLL pretreatment, demonstrating that direct contact between MB49 cells and the urothelium was sufficient to induce UCCM (Figure 4A). Moreover, i.v. administration of PF-573228 efficiently abolished UCCM induced by intravesical MB49 instillation without PLL pretreatment (Figure 4B). Bladders were harvested 6.5 h after intravesical treatment with either vehicle or MB49, and the urothelium was meticulously separated from the underlying connective tissue. RNA was immediately extracted from three experimental groups: vehicle, UCCM (MB49-treated without PLL pretreatment), and UCCM-inhibition (MB49-treated without PLL pretreatment, but treated with i.v. PF-573228 6 h after intravesical MB49 instillation to inhibit UCCM).

Principal-component analysis and transcription heatmaps revealed distinct alterations in the UCCM group compared with the vehicle group. In contrast, differences between the UCCM and UCCM-inhibition groups were modest (Figures 4C and 4D). Gene set enrichment analysis (GSEA) demonstrated the enrichment of gene sets associated with “positive regulation of cell migration” and “cell chemotaxis” in the UCCM group. This enrichment was attenuated in the UCCM-inhibition group (Figure 4E). Gene ontology (GO) biological processes analysis revealed significant enrichment of pathways related to “positive regulation of innate immune response,” “response to LPS,” and “cell migration” in the UCCM group (Figure 4F). Kyoto Encyclopedia of Genes and Genomes (KEGG) pathway analysis further revealed associations between UCCM and several innate immune-related pathways, including NOD-like receptor signaling, TNF signaling, NF-κB signaling, chemokine signaling, and TLR signaling pathways (Figure 4G). Additionally, GSEA highlighted significant upregulation of gene sets related to “innate immune response,” “cellular response to LPS,” “regulation of defense response,” “response to bacterium,” and “positive response of cell migration” (Figure 4H). Given that the urothelium glides collectively over the ECM while maintaining cell-cell contacts during UCCM, we propose the term “urothelial collective-gliding response (UCGR)” to describe this UCCM-associated defensive mechanism.

### UCGR functions as a defense mechanism against bacterial bladder infection

**Figure 5:**
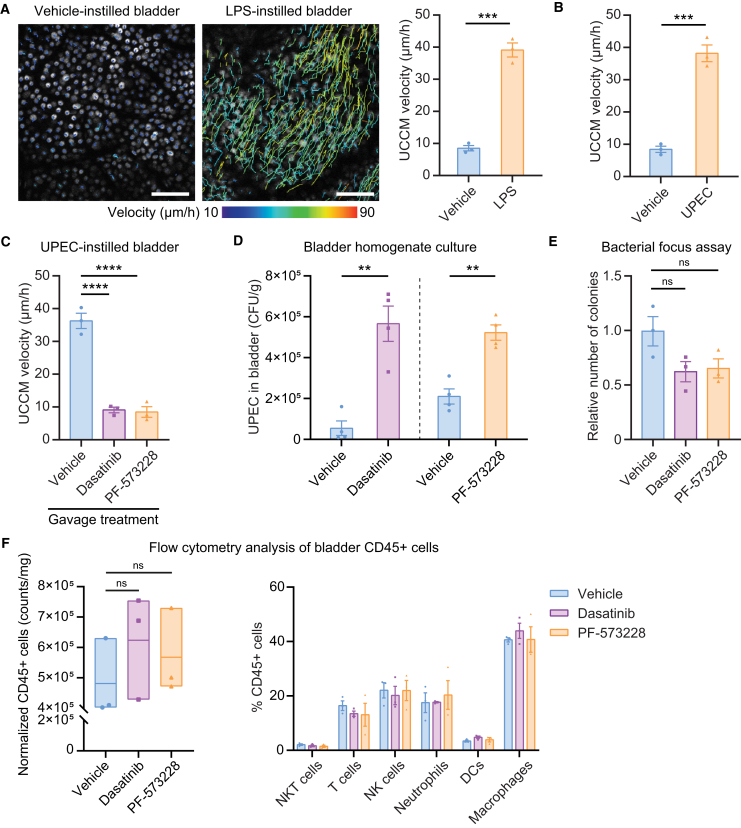
UCGR functions as a defense mechanism against bacterial bladder infection (A) Representative urothelial tracking images and mean UCCM velocity in vehicle- and LPS-instilled bladders 4 h post intravesical treatment. Urothelial cell migration trajectories during 2-h intravital imaging are shown as color-coded lines representing velocities. Scale bars: 100 μm. *n* = 3 mice/group. (B) Mean UCCM velocity in the vehicle- and J96 (UPEC)-instilled bladders 4 h post intravesical treatment. *n* = 3 mice/group. (C) Mean UCCM velocity in the UPEC-instilled bladders treated with oral gavage of vehicle, dasatinib (Src inhibitor), or PF-573228 (FAK inhibitor) followed by intravital imaging 4 h after. *n* = 3 mice/group. (D) UPEC CFU counts per gram of bladder tissue treated with vehicle or dasatinib and vehicle or PF-573228. *n* = 4 mice/group. (E) Colony formation units of UPEC plated on LB-agar plate containing vehicle, dasatinib, or PF-573228. Data normalized to vehicle group. *n* = 3 mice/group. (F) Analysis of immune cells in UPEC-instilled bladders, after a 30-min UPEC instillation and 6 h post-gavage with vehicle, dasatinib, or PF-573228: quantification of live CD45^+^ cells per mg of bladder tissue and percentage distribution of immune cell types. Floating bars indicate minimum, mean, and maximum values. The cell numbers were normalized as described in methods. *n* = 3 mice/group. Data are presented as the mean ± SEM. Unpaired two-sided student’s t test (A, B, and D) and one-way ANOVA with Dunnett’s multiple comparisons test (C, E, and F) ∗∗*p* < 0.01, ∗∗∗*p* < 0.001, ∗∗∗∗*p* < 0.0001.

The RNA-seq results prompted an investigation into the urothelial response to LPS. Intravital imaging of the mouse urothelium 4 h after intravesical LPS instillation revealed directional UCCM, resembling the response to MB49 (Figure 5A). Since LPS is a key component of gram-negative bacterial outer membranes, we examined the UPEC strain J96, which initiated UCCM 4 h after intravesical instillation (Figure 5B). We evaluated the effects of oral gavage of dasatinib and PF-573228 on UPEC-induced UCCM and found that both inhibitors reduced the velocity by approximately 75% at 4 h post-treatment (Figure 5C). Given that dasatinib and PF-573228 inhibition of UCCM promoted MB49 bladder tumor growth, we investigated whether UCCM inhibition exacerbates UPEC-induced bladder infections. Bladder homogenate cultures obtained after intravesical UPEC instillation and gavage with vehicle, dasatinib, or PF-573228 exhibited significantly higher colony counts in inhibitor-treated groups compared to vehicle controls, indicating that UCCM inhibition worsened bacterial bladder infection (Figure 5D). *In vitro* cultures of UPEC on Luria-Bertani (LB)-agar plates demonstrated that neither dasatinib nor PF-573228 significantly promoted UPEC proliferation; instead, both displayed a tendency to reduce proliferation (Figure 5E). Flow cytometric analysis, employing the gating strategy outlined in Figure S4, revealed that these inhibitors did not alter the total number of immune cells or their subtype distribution in UPEC-instilled bladders at 6 h post-gavage (Figure 5F). Collectively, these findings establish the UCGR as a crucial immunological defense mechanism against various bladder pathologies, including tumorigenesis and bacterial infection.

### Urothelium initiates UCGR via TLR4 recognition of immunogenic substances

**Figure 6:**
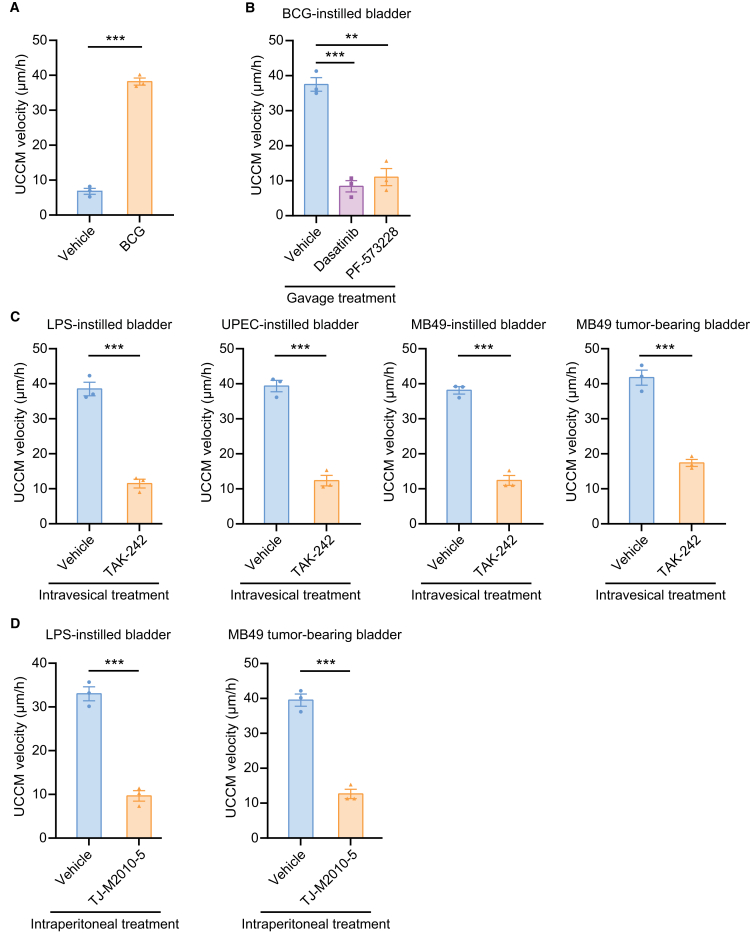
Urothelium initiates UCGR via TLR4 recognition of immunogenic substances (A) Mean UCCM velocity comparing vehicle- and BCG-instilled bladders at 4 h post intravesical treatment. *n* = 3 mice/group. (B) Mean UCCM velocity in BCG-instilled bladders at 4 h post-oral gavage with the vehicle, dasatinib (Src inhibitor), and PF-573228 (FAK inhibitor). *n* = 3 mice/group. (C) Mean UCCM velocity in LPS-, UPEC-, and MB49-instilled bladders and MB49 tumor-bearing bladders at 2 h post-treatment of vehicle and TAK-242. Refers to methods for detailed TAK-242 treatment protocol. *n* = 3 mice/group. (D) Mean UCCM velocity in LPS-instilled and MB49 tumor-bearing bladders at 2 h post i.p. injection of vehicle and TJ-M2010-5 (MyD88 inhibitor). MB49 tumor-bearing mouse experiments were performed 3 days after intravesical instillation of MB49 with PLL pretreatment. *n* = 3 mice/group. Data are presented as mean ± SEM. Unpaired two-sided student’s t test (A, C, and D) and one-way ANOVA with Dunnett’s multiple comparisons test (B). ∗∗∗*p* < 0.001.

Both mouse and human urothelial cells express the innate immune receptor, TLR4,^28^^,^^29^ which recognizes both LPS and UPEC.^30^ Bacillus Calmette-Guérin (BCG), widely used intravesically for treating bladder carcinoma *in situ* and preventing bladder tumor recurrence following transurethral resection of bladder tumor (TURBT),^31^ is also recognized by TLR4, triggering an innate immune response.^32^ We found that intravesical instillation of the BCG Tokyo-172 strain initiated UCCM (Figure 6A) and oral gavage of dasatinib and PF-573228 reduced UCCM (Figure 6B), further supporting the role of TLR4 in this response.

To further confirm the role of TLR4, we test the selective TLR4 inhibitor TAK-242. TAK-242 robustly suppressed UCCM induced by intravesical instillations of MB49 (without PLL pretreatment), LPS, and UPEC, reducing UCCM velocity by 68%, 70%, and 69%, respectively (Figure 6C). TAK-242 also significantly reduced UCCM velocity by 58% in MB49 tumor-bearing mouse bladders (Figure 6C). Inhibition of MyD88, a TLR4 downstream molecule, by TJ-M2010-5 further reduced UCCM velocity by >70% in both LPS-instilled and MB49 tumor-bearing urothelia (Figure 6D). These findings demonstrate that immunogenic substances trigger UCCM through TLR4 recognition in the urothelium, inducing UCGR as a defense mechanism against bladder pathologies.

### Matrix metalloprotease (MMP)-8 and MMP-9 from urothelium initiate UCCM, and their enhancement of UCGR reduces bladder tumor growth and bacterial bladder infection

**Figure 7:**
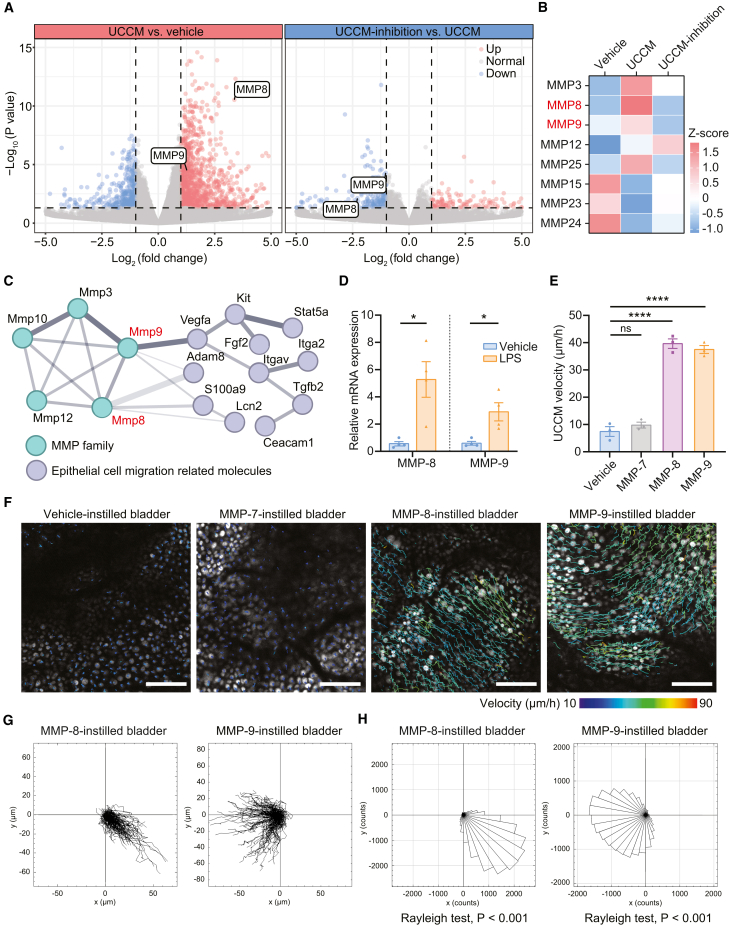
MMP-8 and MMP-9 from urothelium initiate UCCM (A) Volcano plot comparing DEGs between UCCM group vs. vehicle groups and UCCM-inhibition vs. UCCM groups. Vertical lines delineate log_2_ (fold change) boundaries at −1 and 1; horizontal lines display the statistical significance threshold (*p* < 0.05). Blue and red dots indicate down- and upregulated genes, respectively. (B) Heatmap showing standardized mRNA abundance values (Z scores) of MMP family molecules in the vehicle, UCCM, and UCCM-inhibition groups. Only molecules with significant differences in expression are shown. (C) Protein-protein interaction networks of differentially expressed MMP family proteins and epithelial cell migration-related proteins, highlighting MMP-8 and MMP-9 as two hub genes. (D) RT-qPCR analysis of relative mRNA expressions of MMP-8 and MMP-9 in urothelium 4 h post intravesical instillation of vehicle or LPS. *n* = 4 mice/group. (E) Quantification of mean UCCM velocity in vehicle-, MMP-7, MMP-8, and MMP-9-instilled bladders 4 h post intravesical treatment. *n* = 3 mice/group. (F) Representative urothelial tracking images in MMP-8 and MMP-9-instilled bladders. The migration trajectories of urothelial cells during 2-h intravital imaging are represented by color-coded lines corresponding to migration velocities. (G and H) Cell trajectory analysis showing the nuclear centroid displacement during 2-h imaging (G) and rose diagrams illustrating directional UCCM in MMP-8- and MMP-9-instilled bladders (H) in representative experiments. Scale bars: 100 μm. Data are presented as the mean ± SEM. Unpaired two-sided student’s t test (D), one-way ANOVA with Dunnett’s multiple comparisons test (E) and Rayleigh test (H). ∗*p* < 0.05, ∗∗∗∗*p* < 0.0001.

To identify the molecular triggers of UCGR, we transferred urine from MB49 bladder tumor-bearing mice to the bladders of untreated mice (Figure S5A). Intravital imaging revealed UCCM 4 h after the transfer of urine from MB49 tumor-bearing mice, whereas urine from untreated mice did not induce UCCM (Figure S5B). These results suggest that UCGR is triggered by secreted urothelial factors. Heat denaturation of urine (90°C for 5 min) abolished UCCM, indicating that UCGR-triggering molecules are likely proteins (Figure S5B). Mass spectrometry analysis revealed an enrichment of MMP-8 and MMP-9 in the urine of MB49 tumor-bearing mice compared to controls, although these differences were not statistically significant (Figure S5C). RNA-seq analysis identified hub genes involved in UCGR initiation. A volcano plot of differentially expressed genes (DEGs) revealed a significant upregulation of MMP-8 and MMP-9 in the UCCM group, which was absent in the UCCM-inhibition group (Figure 7A). Heatmap analysis of MMP family expression profiles illustrated selective upregulation of MMP-8, -9, and 25 in the UCCM group, which was abolished in the UCCM-inhibition group (Figure 7B). Protein-protein interaction analysis revealed associations between MMP family proteins and epithelial cell migration-related proteins. Notably, MMP8, MMP9, and MMP3, which are known to activate MMP-8 and MMP-9, were strongly connected with epithelial cell migration regulators, such as ADAM8, KIT, and STAT5A (Figure 7C).^33^^,^^34^^,^^35^ Reverse transcription quantitative PCR (RT-qPCR) analysis confirmed the upregulation of MMP-8 and MMP-9 in the LPS-treated urothelium compared to vehicle-treated controls (Figure 7D). Together, these results suggest that MMP-8 and MMP-9 play a crucial role in UCGR triggered by various immunogenic substances.

**Figure 8:**
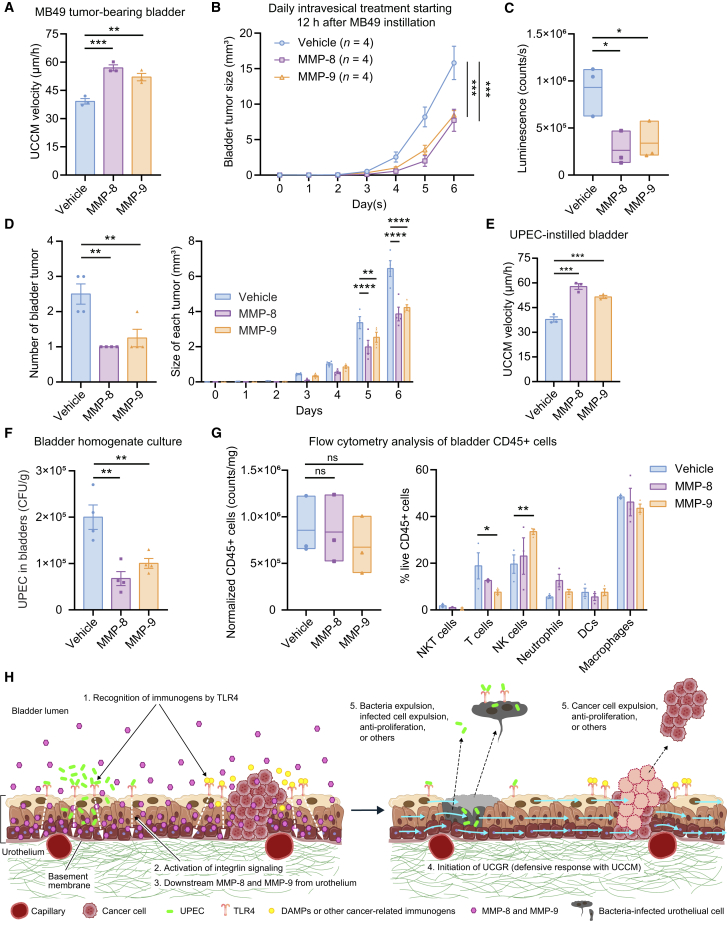
UCGR enhanced by MMP-8 and MMP-9 reduces MB49 tumor growth and UPEC bladder infection (A–C) Impact of intravesical instillation of MMP-8 and MMP-9 on UCCM and MB49 bladder tumor growth. (A) Mean UCCM velocity in MB49 tumor-bearing bladders 4 h post intravesical instillation of vehicle, MMP-8, or MMP-9. The imaging experiments were performed 3 days after intravesical instillation of MB49 with PLL pretreatment. *n* = 3 mice/group. (B) MB49 tumor volumes evaluated with ultrasound imaging in mouse bladders treated with daily intravesical instillation of vehicle, MMP-8, or MMP-9. *n* = 4 mice/group. (C) Akaluc/AkaLumine bioluminescence quantification at day 9 post-MP49 instillation (with PLL pretreatment) under daily treatment with vehicle, MMP-8, or MMP-9 instillation. Floating bars indicate the minimum, mean, and maximum values. *n* = 3 mice/group. (D) Quantification of the number of bladder tumors in each mouse and the size of each tumor, using the same mice analyzed in B. The number of bladder tumors was evaluated with ultrasound imaging 4 days post intravesical instillation of MB49 with PLL pretreatment. (E) Mean UCCM velocity in UPEC-instilled bladders 4 h post intravesical instillation of vehicle, MMP-8, or MMP-9. *n* = 3 mice/group. (F) J96 (UPEC) CFU counts per gram of bladder tissue treated with vehicle, MMP-8, or MMP-9. *n* = 4 mice/group. (G) Analysis of bladder immune cells in UPEC-instilled bladders, after a 30-min UPEC instillation and 6 h post-instillation of vehicle, MMP-8, or MMP-9: quantification of live CD45^+^ cells per mg of bladder tissue and the percentage distribution immune cell types. Floating bars indicate the minimum, mean, and maximum values. Cell numbers were normalized as described in methods. *n* = 3 mice/group. (H) Schematic diagram illustrating the potential mechanism of UCGR as a TLR-associated defense mechanism. Data are presented as the mean ± SEM. One-way ANOVA with Dunnett’s multiple comparisons test (A, C, D, E, F, and G) and two-way ANOVA with Dunnett’s test (B). ∗*p* < 0.05, ∗∗*p* < 0.01, ∗∗∗*p* < 0.001, ∗∗∗∗*p* < 0.0001.

Using intravital imaging, we demonstrated that intravesical instillation of recombinant MMP-8 or MMP-9 individually initiated UCCM with a velocity of approximately 40 μm/h. In contrast, neither vehicle nor another subclass of MMP, MMP7, induced UCCM (Figure 7E). Cell migration tracking analysis confirmed that both MMP-8- and MMP-9-induced UCCM exhibited directional migration characteristics (Figures 7F–7H). Interestingly, intravesical instillation of MMP-8 and MMP-9 accelerated the velocity of MB49 tumor-induced UCCM by 46% and 33%, respectively (Figure 8A). Based on these results, we investigated whether MMP-8- and MMP-9-enhanced UCGR suppresses MB49 tumor development. Following PLL pretreatment and MB49 instillation, mice received daily intravesical instillations of MMP-8, MMP-9, or vehicle. Both MMP-8 and MMP-9 treatment groups showed reduced MB49 tumor growth compared to vehicle controls by approximately 50% (Figure 8B). This observation was confirmed by bioluminescence imaging (Figure 8C). Notably, both the number of tumors and the size of each tumor were reduced by MMP-8 and MMP-9 (Figure 8D).

In the bacterial infection studies, mice were intravesically instilled with UPEC for 30 min, followed by 1 h of intravesical treatment with MMP-8, MMP-9, or vehicle. We confirmed that intravesical instillation of MMP-8 and MMP-9 also accelerated the velocity of UPEC-induced UCCM by 52% and 39%, respectively (Figure 8E). Bladder homogenate cultures assessed 6 h after UPEC treatment showed fewer colonies in MMP-treated bladders compared to vehicle controls (Figure 8F). These results further confirm the defensive role of UCGR against both bladder tumor growth and bacterial bladder infection.

Finally, we evaluated the effects of MMP-8 and MMP-9 on immune cells, particularly those involved in innate immunity, using flow cytometry (gating strategy shown in Figure S4). In UPEC-instilled bladders, neither MMP-8 nor MMP-9 altered the total number of immune cells or their subtype distribution, except for a significant decrease in T cells and an increase in NK cells observed in MMP-9-instilled bladders compared with vehicle controls. This suggests that neither MMP-8 nor MMP-9 significantly affected the overall innate immune status (Figure 8G), and their primary effects were on urothelial cells.

## Discussion

In this study, we discovered that the urothelium responds to a variety of immunogenic substances, including UPEC, LPS, BCG, and bladder cancer cells, by collectively gliding over the ECM, a phenomenon we termed “UCCM.” Inhibition of UCCM enhanced the growth of MB49 bladder tumors (Figure 3) and exacerbated UPEC bladder infection (Figure 5). At the same time, UCCM initiation suppressed these pathological processes (Figure 8). The MB49-treated urothelium exhibited upregulation of genes related to the innate immune response and cell migration. Inhibition of the innate immune receptor TLR4 blocked UCCM (Figure 6). These findings highlight the protective role of UCCM within the TLR4-associated response to immunogenic substances, leading us to designate this process as the “UCGR” (Figure 8H).

To investigate the mechanism underlying UCCM induction, we performed RNA-seq comparing the control urothelium with the MB49-treated urothelium without PLL pre-treatment. This condition was chosen because it was expected to cause less acute inflammation than treatments with UPEC, LPS, or BCG. Analysis of DEGs revealed that the MB49-treated urothelium was enriched for migration-related gene sets and also for innate immune-related gene sets. These findings suggest that innate immune receptors are involved in the induction of UCCM. TLR4, a pattern recognition receptor, recognizes LPS^36^ and LPS-presenting Gram-negative bacteria such as *E. coli*. As anticipated, administration of the TLR4 inhibitor, TAK-242, blocked UCCM induced by UPEC and LPS (Figure 6C). Interestingly, TAK-242 also abolished UCCM induced by MB49 (Figure 6C). TLR4 is also activated by damage-associated molecular patterns (DAMPs) upregulated in cancerous lesions.^37^^,^^38^ Thus, TLR4 likely initiates UCCM by recognizing DAMPs from either MB49 cells or the urothelium in MB49-treated bladders.

It has been demonstrated that a single immediate instillation of an anthracycline antitumor agent following TURBT for non-muscle invasive bladder cancer prevents recurrence, becoming a standard treatment.^39^ However, in addition to the direct cytotoxic effects of anthracyclines, there may also be a mode of action where anthracyclines induce immunogenic cell death,^40^ which in turn triggers UCCM and leads to tumor-suppressive effects. Similarly, in the treatment and recurrence prevention of non-muscle-invasive bladder cancer with intravesical BCG treatment, the induction of UCCM may contribute to its tumor-suppressive effects in addition to the well-established immune cell response induced by BCG.^41^ This study did not elucidate the precise mechanism by which the TLR4 activates the integrin signaling pathway. However, previous studies have shown that TLR4 activation induces multiple molecular pathways, some of which lead to the activation of adaptor proteins involved in integrin signaling in immune cells.^42^ Based on these findings, a similar signaling mechanism may operate in the urothelium during UCCM.

Bulk RNA-seq analysis comparing the vehicle and UCCM groups identified MMP-8 and MMP-9 as key initiators of UCCM. In the UCCM-inhibition group, where Src and FAK inhibitors were used, the enrichment of these MMPs was abolished, suggesting that integrin signaling regulates their expression downstream (Figure 7). This aligns with previous findings, as activation of integrin signaling has been shown to upregulate MMPs, including MMP-8 and MMP-9^18^^,^^43^. MMPs are zinc-containing endopeptidases that degrade ECM and are involved in various processes, such as cytokine production, wound healing, cancer invasion, and metastasis.^44^ MMP-8 and MMP-9 play diverse roles in cancer development and progression.^45^^,^^46^^,^^47^^,^^48^ Previous studies have demonstrated that MMP-8 works synergistically with MMP-9 during keratinocyte migration during wound closure.^49^ Moreover, MMP-9 regulates cell migration by degrading ECM components, such as collagens and laminin.^50^ It can also enhance epithelial cell migration through MAPK-dependent pathways, independent of its proteolytic activity.^50^ We demonstrated that intravesical instillation of MMP-8 or MMP-9 induced UCCM and enhanced the defense mechanism of UCGR, suggesting potential therapeutic strategies by activating UCGR. For instance, intravesical instillation of MMP-8 and MMP-9 could help eliminate antibiotic-resistant UPEC or support the efficacy of antibiotics. Since intravesical MB49 instillation following PLL pretreatment serves as a model for bladder cancer recurrence after TURBT,^51^ intravesical instillation of MMP-8 and MMP-9 post-TURBT may offer a strategy to prevent bladder tumor recurrence. However, given the dual role of MMPs in promoting both tumor suppression and progression, their therapeutic applications require careful evaluation, particularly in the context of cancer treatment.

We validated the anti-bladder tumor effects of UCCM and UCGR using two approaches. First, we compared the effects of UCCM inhibition on MB49 bladder cancer cell growth using orthotopic and subcutaneous implantation models. Daily oral gavage of dasatinib and PF-573228 enhanced tumor growth in the bladder but not in the subcutaneous tissue, indicating that their effects are specific to the orthotopic model. Notably, both inhibitors suppressed MB49 tumor growth *in vitro* (Figure S3B), suggesting that their tumor-enhancing effects *in vivo* are mediated through UCCM inhibition. Consistent with the subcutaneous implantation model, dasatinib has been reported to exhibit antitumor effects against bladder cancer cells.^52^^,^^53^ The impact of PF-573228 on bladder cancer cells remains unclear. However, FAK inhibition has been reported to suppress migration, invasion, and proliferation of bladder cancer cells.^54^^,^^55^ Secondly, we enhanced UCCM with MMP-8 and MMP-9 instillation. As anticipated, this treatment significantly suppressed both MB49 tumor growth and UPEC bladder infection (Figure 8). These findings support the notion that UCCM functions as a TLR4-associated defense mechanism in the bladder, in addition to previously known TLR4-initiated innate immune response.

Our study represents a significant advancement in the identification and partial elucidation of previously unrecognized biological phenomena. Previous studies have shown that various TLRs are expressed in the urothelium and participate in processes such as immune cell-attracting cytokine secretion and afferent nerve stimulation^56^^,^^57^; however, their precise significance remains unclear. Our study revealed a novel function of the TLR4 innate immune receptor in the urothelium, where it mediates UCGR as a defense mechanism. This is the first demonstration that mature epithelial cells initiate CCM in response to specific substances through receptor-mediated recognition, either in the urothelium or other tissues. Further investigation into the mechanisms of UCGR and the development of methods to modulate this response could lead to novel therapeutic strategies for various bladder pathologies. Furthermore, similar collective epithelial migration mechanisms may occur in other organs and warrant further investigation.

### Limitations of the study

Our study had some limitations. First, stable intravital imaging and intravesical drug instillation necessitate the regulation of bladder pressure via urinary catheterization. Due to anatomical constraints, transurethral catheterization is not feasible in male mice,^58^ limiting our study to female mice. To our knowledge, the molecules and signaling pathways involved in the mechanism of UCCM shown in this study have not been reported to differ between sexes and are therefore expected to occur similarly in males. Nevertheless, caution should be exercised when generalizing the findings demonstrated in this study to male mice. Second, this study has elucidated only part of the mechanisms underlying UCCM and UCGR. For example, it does not clarify how the recognition of immunogenic substances by TLR4 leads to the activation of integrin signaling, nor how integrin signaling results in the upregulation of MMP. Furthermore, the precise mechanism by which UCGR eliminates pathogens remains unclear. Mechanical stress induced by UCCM may affect cancer cell proliferation or induce apoptosis or extrusion via mechanisms such as cell competition,^59^ which warrants further investigation. Third, whether UCGR occurs in humans remains uncertain. Current technological limitations prevent the verification of this phenomenon in human tissues, highlighting the need for advanced imaging techniques.

## Resource availability

### Lead contact

Further information and requests for resources or reagents should be directed to and will be fulfilled by the lead contact: Takeshi Sano (sanotake@kuhp.kyoto-u.ac.jp).

### Materials availability

No materials such as reagents or other products were generated in this study.

### Data and code availability

• Data: Raw data underlying the figures have been deposited in Mendeley data and are publicly available at https://data.mendeley.com/preview/7c6k563fdc?a=bffcbff1-9890-4a64-9236-e6e6ed1edeb9. The bulk RNA-seq data have been deposited in the NCBI Sequence Read Archive (accession number: PRJNA1227871), and the proteomics data have been deposited in ProteomeXchange via jPOST (accession number: PXD061318). Both datasets are publicly available as of the date of publication.
• Code: This paper does not report original code.
• Additional information: Any additional information required to reanalyze the data reported in this paper is available from the lead contact upon request.

## Acknowledgments

We extend our gratitude to Margaret A. Knowles for providing the UMUC-3 cell line utilized in this study. We thank Ms. Kanako Takakura (Kyoto University) for her technical assistance, Ms. Haruka Mii (Kyoto University) for support with the randomized mouse study, Ms. Eriko Deguchi (Kyoto University) for processing the RNA-seq data, and Dr. Kenta Terai (Tokushima University) for valuable discussions regarding study design and RNA-seq analysis. This work was funded by a grant-in-aid for early-career scientists from the Japan Society for the Promotion of Science (20K18137 to T. Sano), a GSK Japan Research grant 2020 (to T. Sano), a JST Moonshot R&D grant (JPMJPS2022 to M.M.), and a Young Researcher Promotion grant from the Japanese Urological Association (to T. Sano).

## Author contributions

Data curation, formal analysis, investigation, methodology, software, validation, visualization, writing – original draft: N.Z.; conceptualization, data curation, funding acquisition, investigation, methodology, project administration, visualization, writing – original draft: T. Sano.; formal analysis, investigation, resources, writing – original draft: K.I.; investigation, resources, writing – original draft: S.I.; writing – review and editing: R.I.; writing – review and editing: H.T.; writing – review and editing: K.N.; writing – review and editing: T. Sakatani.; writing – review and editing: A.H.; formal analysis, writing – review and editing: M.T.; writing – review and editing: K.M.; writing – review and editing: Y.K.; writing – review and editing: T. Sumiyoshi.; writing – review and editing: T.G.; writing – review and editing: R.S.; supervision: O.O. formal analysis, funding acquisition, methodology, resources, supervision, writing – original draft: M.M.; funding acquisition, methodology, resources, supervision, writing – review and editing: T.K.

## Declaration of interests

The authors have no conflicts of interest to declare.

## STAR★Methods

### Key resources table

**Table:** 

REAGENT or RESOURCE	SOURCE	IDENTIFIER
**Antibodies**		

anti-CD45, BUV395	BD Biosciences	Cat# 564279, RRID:AB_2651134
anti-TCR beta chain, BUV737	BD Biosciences	Cat# 612821, RRID:AB_2870145
anti-CD11b, BV480	BD Biosciences	Cat# 553312, RRID:AB_398535
anti-I-A/I-E, BV650	BioLegend	Cat# 107641, RRID:AB_2565975
anti-CD11c, BV785	BioLegend	Cat# 117336, RRID:AB_2565268
anti-NK1.1, PE/Dazzle 594	BioLegend	Cat# 108748, RRID:AB_2564219
anti-Ly-6G, PE/Cy7	BD Biosciences	Cat# 560601, RRID:AB_1727562
anti-CD8a	BioLegend	Cat# 100711, RRID:AB_312750
anti-CD4	BioLegend	Cat# 116021, RRID:AB_2715957
anti-CD16/CD32	BioXCell	Cat# BE0307

**Bacterial and virus strains**		

UPEC strain J96	ATCC	Cat# ATCC-700336
BCG Tokyo-172 strain	Japan BCG Laboratory	N/A

**Biological samples**		

RNA extracted from the urothelium of EKAREV-NLS mice	This paper	N/A
Urine collected from EKAREV-NLS mice	This paper	N/A

**Chemicals, peptides, and recombinant proteins**		

Poly-L-Lysine (PLL) Solution	Sigma-Aldrich	Cat# P4707
Qtracker 655	Thermo Fisher Scientific	Cat# Q25021MP
PD0325901	EMD Millipore	Cat# 444966
Dasatinib	AdooQ BioScience	Cat# A10290
PF-573228	Selleck Chemicals	Cat# S2013
Lipopolysaccharide (LPS) Solution	Thermo Fisher Scientific	Cat# 00-4976-93
TAK-242	MedChemExpress	Cat# HY-11109
TJ-M2010-5	MedChemExpress	Cat# HY-139397
Recombinant MMP-7	R&D systems	Cat# 2967-MP-010
Recombinant MMP-8	R&D systems	Cat# 2904-MP
Recombinant MMP-9	R&D systems	Cat# 909-MM-010
AkaLumine luminescent substrate	Iwano et al.^60^	N/A

**Critical commercial assays**		

RNeasy Mini Kit	QIAGEN	Cat# 74104
ReverTra Ace qPCR RT Kit	Toyobo	Cat# FSQ-101
Zombie NIR™ Fixable Viability Kit	BioLegend	Cat# 423105

**Deposited data**		

RNA sequencing data	NCBI	PRJNA1227871
Mass spectrometry proteomics	ProteomeXchange	PXD061318
Raw data underlying the figures	This paper	Mendeley Data: https://data.mendeley.com/preview/7c6k563fdc?a=bffcbff1-9890-4a64-9236-e6e6ed1edeb9

**Experimental models: Cell lines**		

MB49	Sigma-Aldrich	Cat# SCC148
UMUC-3	Margaret A. Knowles (St. James University Hospital)	N/A

**Experimental models: Organisms/strains**		

Mouse: EKAREV-NLS	Kamioka et al.^17^	N/A

**Oligonucleotides**		

GAPDH forward 5'-CATCACTGC CACCCAGAAGACTG-3’	This paper	N/A
GAPDH reverse 5'-ATGCCAGT GAGCTTCCCGTTCAG-3'	This paper	N/A
MMP-8 forward 5'-TCTTCCTCC ACACACAGCTTG-3’	This paper	N/A
MMP-8 reverse 5'-CTGCAACC ATCGTGGCATTC-3'	This paper	N/A
MMP-9 forward 5'-CTGGACAG CCAGACACTAAAG-3’	This paper	N/A
MMP-9 reverse 5'-CTCGCGGC AAGTCTTCAGAG-3’	This paper	N/A

**Recombinant DNA**		

pCSIIhyg-H2B-mScarlet-P2Av3-akaluc	Iwano et al.^60^	N/A
pCMV-VSV-G-RSV-Rev	RIKEN BioResource Center	Cat# RDB04393
psPAX2	Addgene	Cat#12260

**Software and algorithms**		

GraphPad Prism (v9.4.1)	GraphPad	http://www.graphpad.com/
FlowJo (v10)	BD Biosciences	https://www.flowjo.com
R studio (v2023.06.0 + 421)	R studio	https://posit.co/downloads/
GSEA desktop tool (v4.3.2)	Broad Institute	https://www.gsea-msigdb.org/gsea/index.jsp
MetaMorph (v.7.8.0.0)	Molecular Devices	https://www.moleculardevices.com.cn/
ImageJ (v.1.54f)	NIH	https://imagej.nih.gov/ij/
Chemotaxis & Migration Tool (v 1.01)	ibidi GmbH	https://ibidi.com/chemotaxis-analysis/171-chemotaxis-and-migration-tool.html
STRING (v11.5)	STRING Consortium	https://string-db.org/
Living Image (v.4.5.5)	Perkin Elmer	https://www.perkinelmer.com
FluoView software (v4.1a)	Olympus	http://www.olympusconfocal.com/products/fv1000/fv1000software.html
ProteinPilot software (v5.0.1)	SCIEX	https://sciex.com/products/software/proteinpilot-software
FastQC software (v0.11.9)	Andrews	https://www.bioinformatics.babraham.ac.uk/projects/fastqc/

### Experimental model and study participant details

#### Study and ethics approval

Animal protocols were reviewed and approved by the Animal Care and Use Committee of Kyoto University Graduate School of Medicine Kyoto, Japan (approval numbers: 12064, 13074, 14079, and 15064).

#### Animals

Transgenic mice expressing ERK-FRET biosensors have been described previously.^17^ The ERK FRET biosensor, EKAREV-NLS, is localized in the nucleus. EKAREV-NLS was backcrossed for more than five generations to C57BL/6NJcl (CLEA Japan). To date, no disease or anomaly has been associated with the expression of EKAREV–NLS used in this study. Mice were housed in a specific pathogen-free facility in temperature-controlled rooms with a 12-h light/12-h dark cycle and were provided a routine chow diet and water *ad libitum*. All experiments were conducted using female mice aged 8–10 wk. Mice were euthanized with carbon dioxide inhalation after experiments or a humane endpoint.

#### Cell lines

UMUC-3, a human urothelial bladder cancer cell line, was generously provided by Margaret A. Knowles (St. James University Hospital). MB49 cells (SCC148, Sigma-Aldrich), derived from carcinogen-induced urothelial carcinoma in male C57BL/6 mice,^61^ and UMUC-3 cells were cultured in DMEM supplemented with 10% heat-inactivated FBS and 1% (v/v) penicillin-streptomycin (09367-34, Nacalai Tesque). Cultures were maintained at 37°C in a 5% CO_2_ atmosphere and used for experiments at early passages (<10 passages). Both cell lines tested negative for mycoplasma contamination. For lentiviral production, HEK-293T cells were co-transfected with pCSIIhyg-H2B-mScarlet-P2Av3-akaluc,^60^ psPAX2 (#12260; Addgene), and pCMV-VSV-G-RSV-Rev (RDB04393, RIKEN BioResource Center). Virus-containing media were filtered and collected 48 h after transfection. Target cells were infected in the presence of 10 μg/mL polybrene (12996-81, Nacalai Tesque). Two days post-infection, infected cells were selected using 500 μg/mL hygromycin (31282-04-9, FujiFilm Wako Pure Chemical Corporation).

#### Microbe strains

The UPEC strain J96 (ATCC, ATCC-700336) was originally isolated from a patient with pyelonephritis. J96 harbors both type 1 and P fimbriae, expressed under specific culture conditions.

#### Orthotopic bladder cancer mouse model

Bladder cancer culture cells were detached from cell culture dishes using 0.05% Trypsin EDTA (25-300-054, Gibco) at 37°C for 3 min. The cells were subsequently washed twice with DMEM, and their viability was assessed using a Countess II automated cell counter (Thermo Fisher Scientific). Cells were resuspended in DMEM at a concentration of 1 × 10^6^ cells per 20 μL. For intravesical instillation, a 24-gauge ethylene tetrafluoroethylene catheter (Terumo) was employed to introduce the cell resuspension into 8–10-wk-old female mice. Prior to administering the cell resuspension, 20 μL of 0.01% PLL (P4707, Sigma-Aldrich) and 80-μL air were instilled into the bladder of anesthetized mice and maintained for 20 min. Afterward, bladder contents were expelled by gently applying pressure to the lower abdomen. Subsequently, 20 μL of the tumor cell mixture and 80-μL air were instilled into the bladder and maintained for 1.5 h. Throughout the procedure, mice were positioned head-down on an 80-degree slope under isoflurane inhalation, with heating pads used to maintain core body temperature. All mice developed bladder tumors at multiple sites, including the bladder dome.

#### Subcutaneous bladder tumor mouse model

MB49 cells (5 × 10^5^) diluted in 100 μL of PBS were subcutaneously injected into the right shaved flank of 8–10 wk-old female mice. Tumor growth was monitored daily using a digital caliper, and tumor volume was calculated using the modified ellipsoidal formula: tumor volume = (width)^2^ × (length))/2.^62^

#### Mouse model of urinary tract infection

J96 cells were statically grown overnight in LB broth prior to infection. Bacteria concentration (bacteria/mL) was determined spectrophotometrically at 600 nm. The J96 suspension was centrifuged at 3600 rpm for 20 min, and the pellet was washed twice with PBS. The final suspension was adjusted in PBS at 1 × 10^8^ CFU per 50 μL. Prior to inoculation, the bladders of anesthetized mice were emptied by gently applying digital pressure. Under aseptic conditions, 50-μL suspension of J96 was instilled into the bladder using a 24-gauge ethylene tetrafluoroethylene catheter (Terumo) and retained for 30 min.

### Method details

#### TPEM

We utilized an FV1000MPE-BX61WI upright microscope (Olympus) equipped with a 25×/1.05 water-immersion objective lens (XLPLN 25XWMP, Olympus) and an InSight DeepSee Ultrafast laser (0.95 W at 900 nm, Spectra Physics). The excitation wavelength for cyan fluorescent protein (CFP) was set to 840 nm. For filtering and detection, we employed an infrared light-cut filter, BA685RIF-3 (Olympus), two dichroic mirrors, DM505 and DM570 (Olympus), and four emission filters: FF01-425/30 (Semrock) for SHG, BA460–500 (Olympus) for CFP, BA520–560 (Olympus) for YFP, and 645/60 (Chroma Technology) for Qtracker 655 (Q25021MP, Thermo Fisher Scientific). Qracker 655 was i.v. administered alongside other reagents to confirm drug delivery to the target organs. The microscope was equipped with a two-channel GaAsP detector unit and two built-in photomultiplier tubes. FluoView software version 4.1a (Olympus) was used for microscope control, and images were saved in a multilayer 16-bit tagged image file format. The recorded images were processed and analyzed using MetaMorph software (Molecular Devices).

#### Intravital imaging

Female mice were anesthetized using isoflurane inhalation and positioned supine on an electric heating pad maintained at 37°C. A 24-gauge ethylene tetrafluoroethylene catheter (Terumo), connected to a 50-mL bottle of normal saline (3311401H3039; Otsuka Pharmaceutical Factory), was inserted transurethrally into the bladder. Intravesical pressure was regulated by adjusting the bottle height and maintaining a pressure of 10–15 cm H_2_O, consistent with the range observed between the urine storage and voiding phases in the mouse bladder.^63^ To facilitate imaging, the bladder was exteriorized through a lower midline incision and immobilized using a custom-made vacuum-stabilized imaging window (Olympus). For multidimensional visualization of the urothelium and underlying ECM, Z-stack images were acquired with a ×1.3 digital zoom at 1.0 μm intervals and at a scan speed of 4.0 μs/pixel. CFP and SHG were utilized to visualize cells and collagen fibers, respectively. Imaging of Qtracker 655 was performed exclusively following its administration during the imaging session. Time-lapse z-stack images were captured every 5 min.

#### Analysis of ERK activity

ERK activity was visualized using the FRET/CFP ratio image as previously described.^64^ FRET level was represented by the FRET/CFP ratio image in the intensity-modulated display mode, with eight colors ranging from red to blue representing the FRET/CFP ratio and 32 grades of color intensity indicating CFP signal intensity. Warm colors corresponded to higher FRET levels, whereas cooler colors represented lower levels. For each cell, the FRET/CFP ratio was quantified by defining a region of interest (ROI) encompassing the nucleus in background-subtracted CFP and FRET images. The average fluorescence intensity within the ROI was determined for both CFP and FRET images, and the resulting FRET/CFP ratio was used to quantify ERK activity.

#### Cell tracking and analysis of migration velocity for time-lapse intravital imaging

To track cell migration, the FRET images were analyzed using the Fiji TrackMate plugin.^65^ UCCM velocity was calculated using TrackMate as the average velocity of each cell over its tracking period of up to 2 h. The tracking data were further processed, and tracking figures were generated using the Chemotaxis & Migration Tool version 1.01 (ibidi GmbH).

#### Bacterial load assessment

Mice were euthanized 6 h post-transurethral inoculation with J96 via carbon dioxide inhalation. Urine was expelled from the bladder prior to euthanasia. The bladders were aseptically excised and placed in tubes containing 1 ml of sterile PBS to remove residual blood and urine. Each bladder was weighed and individually homogenized using glass tissue grinders (Kontes). The resulting homogenates were serially diluted in PBS and cultured on LB agar. Colony-forming units (CFUs) were enumerated the following day, and bacterial load was expressed as CFU/g of bladder tissue.

#### Administrations of drugs

For i.v. administration, 200-μL PBS supplemented with 4-μL Qtracker 655 and 5 mg/kg of PD0325901, MAPK/ERK kinase inhibitor (444966, EMD Millipore), was injected via the retro-orbital sinus. Dasatinib, a Src inhibitor (A10290, AdooQ BioScience), and PF-573228, an FAK inhibitor (S2013, Selleck Chemicals), were each dissolved in 150-μL propylene glycol supplemented with 4-μL Qtracker 655 and injected via the retro-orbital sinus at a dose of 10 mg/kg. For oral gavage, dasatinib (10 mg/kg) and PF-573228 (10 mg/kg) dissolved in 100 μL of deionized water (ddH_2_O) containing 5% Tween 80 were administrated using a stainless-steel feeding tube.

For intravesical instillation, LPS (20 μg in 100-μL PBS; 00-4976-93, Thermo Fisher Scientific) and BCG Tokyo-172 strain (4 × 10^6^ CFU in 100-μL normal saline, Japan BCG Laboratory) were introduced into the bladder and retained for 1 h. TAK-242 (3 mg/kg; HY-11109, MedChemExpress), a TLR4 selective antagonist, was instilled into the bladder for 1 h, followed by instillation of TAK-242 (3 mg/kg) with either LPS (20 μg), J96 (1 × 10^8^ CFU), or MB49 cells (1 × 10^6^). For the treatment of MB49 tumor-bearing bladder, TAK-242 (5 mg/kg) was instilled into the bladder for 2 h. TAK-242 and all its mixtures were dissolved in 100 μL of PBS containing 5% Tween 80. TJ-M2010-5 (50 mg/kg in 100 μL of PBS; HY-139397, MedChemExpress), a MyD88 inhibitor, was administered i.p.

Recombinant MMPs were activated according to the manufacturer's instructions before use. Recombinant MMP-7 (rMMP-7; 2967-MP-010, R&D systems), rMMP-8 (2904-MP, R&D systems), and rMMP-9 (909-MM-010, R&D systems) were intravesically instilled at a dose of 200 ng, each dissolved in 100 μL of PBS.

#### Colony formation assay

MB49 cells (1 × 10^3^) were seeded into each well of a 6-well plate with 2 mL of DMEM. Dasatinib or PF-573228 was added at various concentrations 24 h after seeding. Cell proliferation was determined 7 days after treatment by fixing the cells with 4% paraformaldehyde (163-20145, Wako) and staining with 0.25% crystal violet (031-04852, Wako) in methanol. The number of colonies was quantified using ImageJ software (NIH).

For bacterial colony formation, J96 suspension (100 CFU) was diluted in 50 μL of PBS and seeded onto an LB agar plate. Vehicle 200 nM dasatinib, or 250 nM PF-573228, was added to LB broth to prepare LB agar containing the respective reagents. The number of CFU on each plate was counted using ImageJ software (NIH) 24 h after seeding.

#### Ultrasonic imaging acquisition and analysis

Mice were anesthetized by isoflurane inhalation, and the hair on their lower abdomen was removed by shaving and applying a chemical hair removal cream. Ultrasound gel (Aquasonic 100, Parker Laboratories) was applied to the abdomen, and the mouse bladders were imaged using a Vevo 2100 High Resolution *In Vivo* Micro Imaging System (Fujifilm VisualSonics). Gross tumor volume was calculated by summing the individual tumor volumes, determined using the modified ellipsoidal formula: tumor volume = (width)^2^ × (length))/2.^62^

#### Bioluminescence imaging

The AkaBLI luciferase-based bioluminescence reporter system, comprising AkaLumine-HCl and Akaluc, provides a light source powerful enough to penetrate body walls, even in deep tissue areas.^60^ Mice bearing MB49-Akaluc tumors in the bladder were administered 100 μL of AkaLumine luminescent substrate, diluted in normal saline, via i.p. injection. The hair on the lower abdomen of the mice was shaved, and the mice were imaged using an IVIS Spectrum *In Vivo* Imaging System (Perkin Elmer) with an exposure time of 30 sec and a view field of 25 cm. Total luminescence counts were quantified by selecting a uniform ROI over the bladder area using the Living Image software package (Perkin Elmer).

#### Proteomics analysis

Proteins were digested with trypsin and purified using an S-Trap micro spin column according to the manufacturer’s instructions (PROTIFI). Liquid chromatography-tandem mass spectrometry analysis of the tryptic digests was performed using a Nano-LC-Ultra 2D-plus liquid chromatograph (Eksigent) coupled with a TripleTOF 5600+ mass spectrometer (SCIEX). A trap column (200 μm × 0.5 mm ChromXP C18-CL 3 μm 120 Å, Eksigent) and an analytical column (75 μm × 15 cm ChromXP C18-CL 3 μm 120 Å, Eksigent) were used for separation. The mobile phase consisted of A  (0.1% formic acid/water) and B  (0.1% formic acid/acetonitrile). The gradient program was as follows: A98%/B2% to A66.8%/B33.2% (125 min), A66.8%/B33.2% to A2%/B98 % (2 min), A2%/B98% (5 min), A2%/B98 to A98%B2% (0.1 min), and A98%/B2% (17.9 min). The flow rate was set to 300 nL/min, and the analytical column temperature was maintained at 40°C. The mass spectrometer was operated in positive electron spray ionization mode, and datasets were acquired using an information-dependent acquisition method. The acquired datasets were analyzed for peptide identification using ProteinPilot software version 5.0.1 (SCIEX) with the UniProtKB/Swiss-Prot database for Mus musculus (April 2022), appended with known common contaminants (SCIEX). The relative abundance of the proteins was estimated using the Progenesis QI platform with Proteomics software version 4.2 (Nonlinear Dynamics). All raw data files were imported to generate aggregates, and peptide identification results with a confidence of at least 95% were used for assignment. Label-free quantification of proteins was performed by relative quantitation using the Hi-N(3) method (Nonlinear Dynamics).

#### Bulk RNA-Seq and gene expression profiling

Total RNA was isolated from the urothelium using the RNeasy Mini Kit (QIAGEN) following the manufacturer’s instructions. RNA quality was assessed using a Nanodrop DS-11 spectrophotometer (DeNovix), ensuring a 260:280 nm ratio ≥ 2.0. RNA integrity was assessed by agarose gel electrophoresis. RNA-Seq analysis was performed on a transcriptome for targeted next-generation sequencing (Macrogen). Total RNA (1 μg) was enriched with polyA + RNA, and sequencing libraries were prepared using the TruSeq stranded mRNA Library kit on the NovaSeq6000 platform (Illumina). RNA-Seq data quality was assessed using FastQC software (v0.11.9). Sequences were aligned to the mus musculus reference genome (GRCm39) using HISAT2 (v2.2.1),^66^ and sequence reads were assigned to reference genomic features using FeatureCounts.^67^ Computational analysis of RNA-Seq data was performed using Galaxy (v22.05.1; Galaxy platform). Gene counts were scaled and normalized to transcripts per kilobase of million (TPM) units. The TPM values of each gene were used to calculate the fold change (FC) and corresponding P values. Differential expression analysis (three biological replicates) was performed using the DESeq2 R package (v1.40.2). Significant DEGs were identified with the criteria of log_2_ FC ≥ 1.0 or log_2_ FC ≤ –1.0 and P < 0.05. A volcano plot was constructed using the ggplot2 R package (v3.4.2). Principal component analysis was performed using the FactoMineR R package (v2.8).

#### Functional enrichment analysis

The DEGs were subjected to GO and KEGG pathway enrichment analyses using the Database for Annotation, Visualization, and Integrated Discovery (DAVID) functional annotation tool (https://davidbioinformatics.nih.gov/).^68^^,^^69^ Enriched GO terms and pathways were selected based on a false discovery rate (FDR) of < 0.05. KEGG pathway and GO were visualized through the clusterProfiler R package (v4.8.2). Heatmaps were generated using the ComplexHeatmap R package (v2.16.0). Protein-protein interaction networks were constructed using STRING (v11.5, https://string-db.org/), where edges represent both functional and physical associations between proteins.

#### GSEA

GSEA was performed using the GSEA desktop tool (v4.3.2).^70^ GSEA analysis was conducted using the clusterProfiler R package (v4.8.2). Enriched phenotypes were considered significant, with a P value of < 0.05 and an FDR of < 0.25. The ranking metric for genes was derived using the "signal-to-noise" method.

#### RT-qPCR

Total RNA was isolated from the urothelium using the RNeasy Mini Kit (QIAGEN) following the manufacturer’s instructions. cDNA was synthesized by a reverse transcriptase reaction with 500 ng of total RNA using the ReverTra Ace qPCR RT Kit (Toyobo). The cDNA was then used as a template for qPCR with the LightCycler 480 SYBR Green I Master (Roche) on a Thermal Cycler Dice Real Time System (Takara Bio). Gene expression levels were normalized to the endogenous control gene GAPDH using the adjusted 2−ΔΔCt method. The primer sequences used for RT-qPCR were as follows: GAPDH: forward 5'-CATCACTGCCACCCAGAAGACTG-3,’ reverse 5'-ATGCCAGTGAGCTTCCCGTTCAG-3'; MMP-8: forward 5'-TCTTCCTCCACACACAGCTTG-3,’ reverse 5'-CTGCAACCATCGTGGCATTC-3'; MMP-9: forward 5'-CTGGACAGCCAGACACTAAAG-3,’ reverse 5'-CTCGCGGCAAGTCTTCAGAG-3.’

#### Flow cytometry

Mice were sacrificed, and whole bladders were transferred to PBS. After weighing and rinsing the bladders with PBS, they were minced and digested at 37°C for 40 min with collagenase IV and DNAse. The cells were filtered through a 70-μL cell strainer and centrifuged at 300× g for 5 min at 4°C. The supernatant was removed, and the cells were suspended in pure FACS buffer. A total of 10 μL of Sphero AccuCount Blank Particles (5.2 μm, Spherotech), corresponding to 10,000 particles, were added to the medium containing the minced urothelium before enzymatic digestion. The counting beads were retained and tracked throughout the sample processing until analysis by flow cytometry. The proportion of beads lost during the process was assumed to be equal to the proportion of cells lost. The absolute cell count (x) was calculated as follows:

Counting beads detected/10,000 (total number of beads added) = number of cells detected/(x). The absolute cell count was normalized to bladder weight (mg).

Samples were stained using the following antibodies: anti-CD45, BUV395 (30-F11, BD Horizon), anti-TCR beta chain, BUV737 (H57-597, BD Horizon), anti-CD11b, BV480 (M1/70, BD Horizon), anti-I-A/I-E, BV650 (M5/114,15.2, BioLegend), anti-CD11c, BV785 (N418, BioLegend), anti-NK1.1, PE/Dazzle 594 (PK136, BioLegend), anti-Ly-6G, PE/Cy7 (1A8, BD Pharmingen), anti-CD8a, APC (53-6.7, BioLegend), anti-CD4, AF700 (RM4-5, BioLegend), and Zombie NIR (BIoLegend). For cell surface staining, the total digested tissue (1–3 × 10^5^ live cells) was diluted with FACS buffer (2% FCS PBS) and blocked with anti-CD16/CD32 (2.4G2, BioXCell) for 20 min. The cells were then stained with appropriately diluted antibodies for 20 min at room temperature. All flow cytometry experiments were performed using a Spectral Cell Analyzer ID7000 (Sony) and analyzed using FlowJo software.

### Quantification and statistical analysis

Data in bar graphs and line graphs are represented as mean ± SEM, and those in violin plots are expressed as median and interquartile range (IQR). The floating bar chart shows the minimum, mean, and maximum values. Paired data are displayed as paired line plots. Prior to the analysis, a normality test was conducted on the collected data. Statistical analyses and data visualization were performed using GraphPad Prism software (v9.4.1, GraphPad) and RStudio software (v2023.06.0 + 421, RStudio) in the R computing environment (v4.3.1). Further information on data presentation and sample numbers is provided in the figure legends.
