# Supplementary material for: Urothelial collective-gliding response acts as a toll-like receptor 4-associated defense mechanism
Source: iScience. 2025 Sep 12;28(10):113553. doi: 10.1016/j.isci.2025.113553 (PMC12508895; doi:10.1016/j.isci.2025.113553)
Supplement: Document S1. Figures S1–S5 [file mmc1.pdf]

iScience, Volume *28*

Supplemental information

Urothelial collective-gliding response acts as a toll-like receptor 4-associated defense mechanism

Ning Zhang, Takeshi Sano, Katsuhiro Ito, Shinji Ito, Ryosuke Ikeuchi, Hideaki Takada, Kenji Nakamura, Toru Sakatani, Akihiro Hamada, Masashi Takeda, Kaoru Murakami, Yuki Kita, Takayuki Sumiyoshi, Takayuki Goto, Ryoichi Saito, Osamu Ogawa, Michiyuki Matsuda, and Takashi Kobayashi

Supplementary figures

Supplemental Figure 1

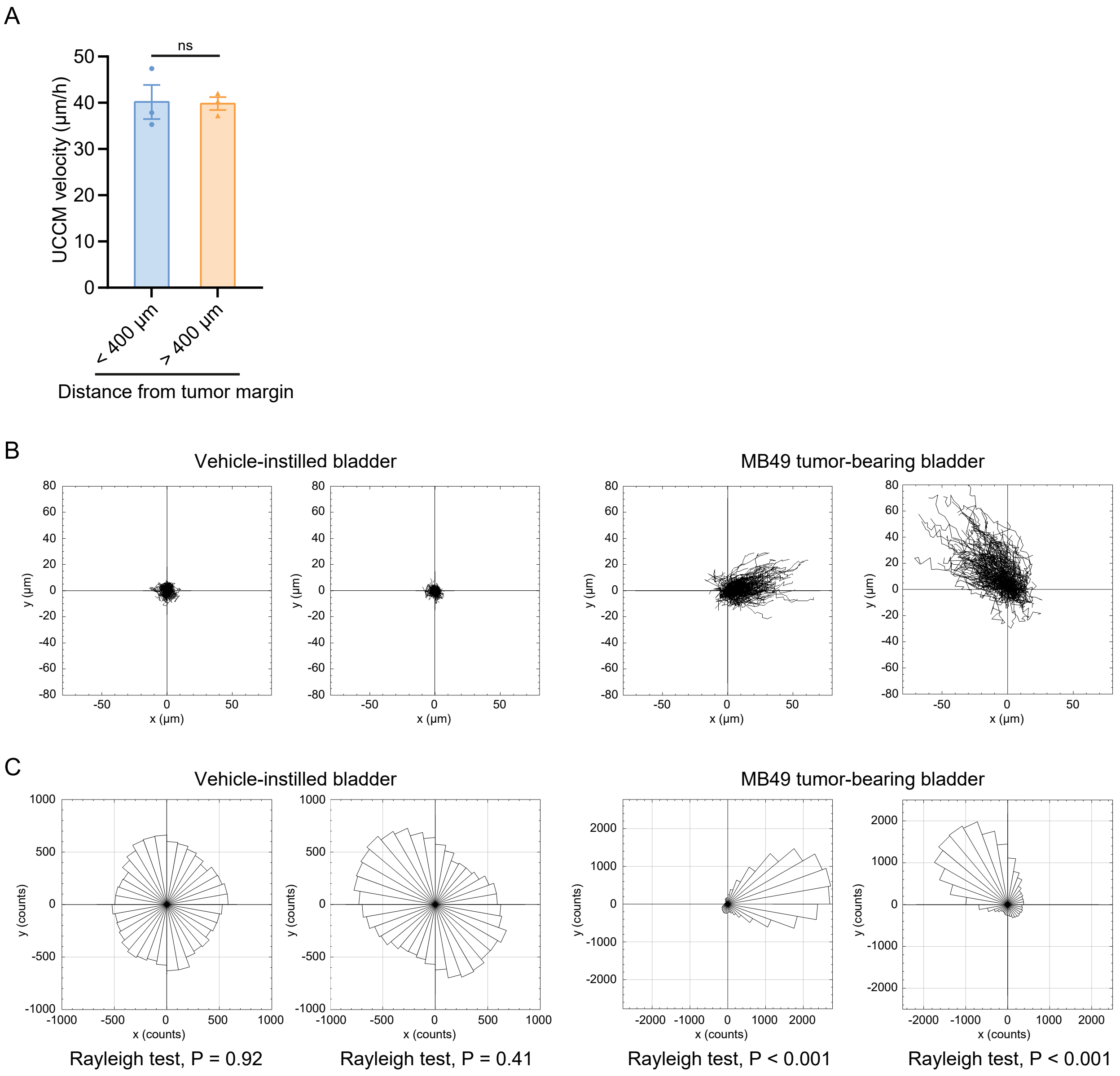

Figure S1. Directional UCCM in MB49 tumor-bearing bladders

(A) Quantification of mean UCCM velocity in MB49 tumor-bearing bladders by 2-h intravital imaging in regions within 400 µm of the tumor margin and those >400 µm away from the tumor margin. n = 3 mice/group. Data are presented as mean ± SEM. Unpaired two-sided Student's t-test. (B and C) Two additional representative experiments from control and MB49 tumor-bearing bladders demonstrating the consistency of findings in Figure 1G and H. (B) Cell trajectory analysis showing nuclear centroid displacement during 2-h imaging in representative experiments. (C) Rose diagrams illustrating the directional preference of urothelial cell migration.

Supplemental Figure 2

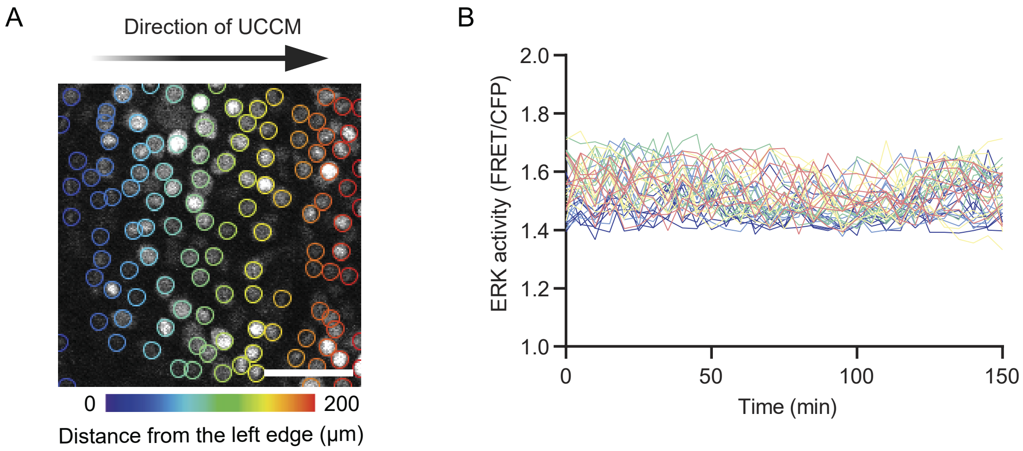

Figure S2. Temporal changes in urothelial ERK activity at different locations from the front to the rear of UCCM

(A) Image showing UCCM at time 0 in MB49-bearing bladders, cropped to exclude areas outside the target cells for analysis. Colors ranging from cool (blue) to warm (red) are assigned to each cell based on its position, from the rear to the front, in the direction of UCCM. (B) Time series of ERK activity (FRET/CFP) in each cell, displayed by the colors assigned in Fig. S2 A, showing no patterns suggesting the propagation of ERK activation.

Supplemental Figure 3

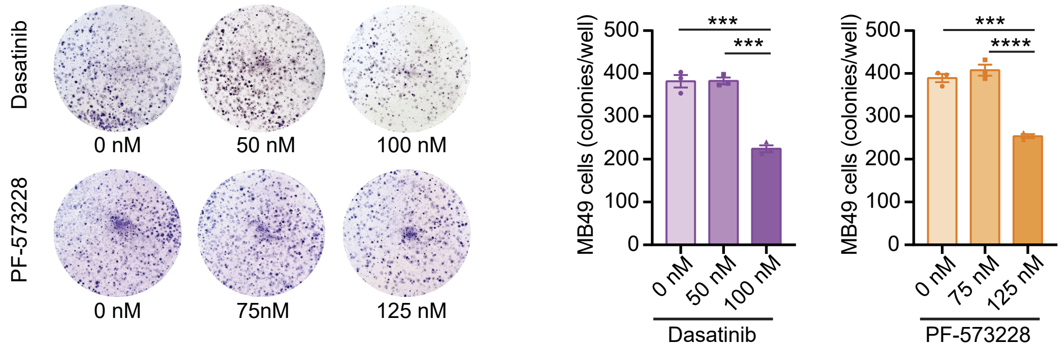

Figure S3. Effects of dasatinib and PF-573228 on MB49 cell growth in vitro

MB49 cell colony formation assay: Photographic representation of a six-well plate seeded with MB49 cells treated with varying concentrations of dasatinib or PF-573228, along-side quantification of colony numbers 7 days post-seeding. $n = 3$ experiments/group. Data are presented as the means ± SEM. One-way ANOVA with Tukey's multiple comparisons test. ***P < 0.001, ****P < 0.0001.

Supplemental Figure 4

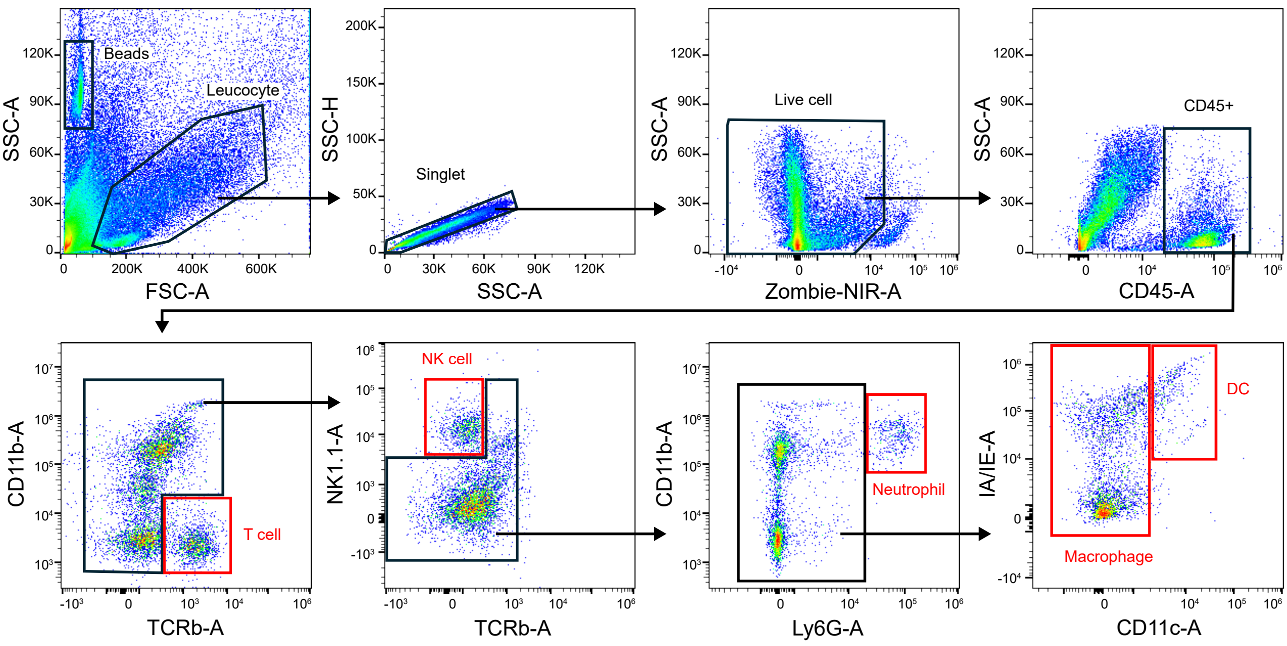

Figure S4. Flow cytometry gating strategy

Flow cytometry gating strategy for the identification and classification of major immune cell populations in the tissues of UPEC-instilled mice treated with intravenous vehicle, dasatinib, and PF-573228, or intravesical vehicle, MMP-8, and MMP-9.

Supplemental Figure 5

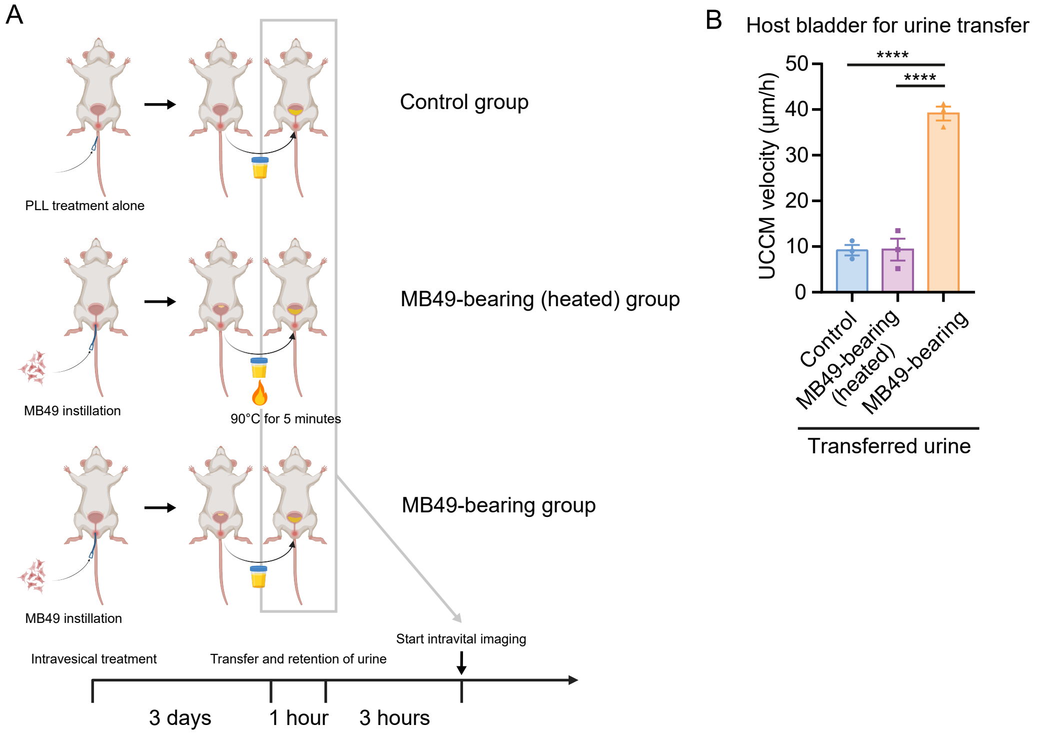

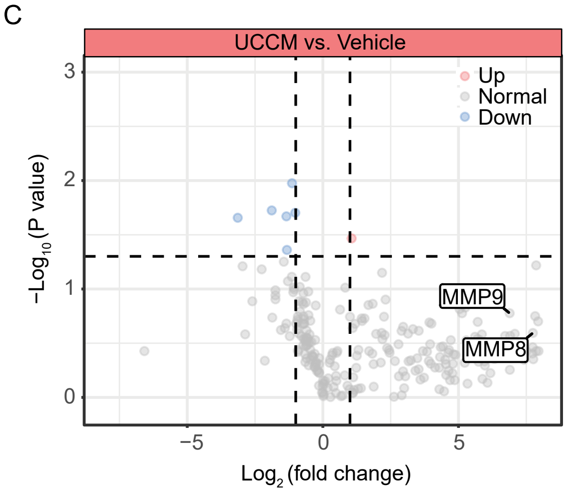

Figure S5. Urine transfer experiments and proteomics analysis

(A) Schematic illustrating the procedure for bladder-to-bladder urine transfer in mice. First, each mouse undergoes either intravesical PLL treatment alone or intravesical PLL treatment followed by MB49 bladder instillation. Three days later, urine from these mice is transferred to the bladders of syngeneic recipient mice and retained for 1 h. After an additional 3 h, intravital imaging is performed (created with BioRender.com). (B) Quantification of mean UCCM velocity in bladders to which urine from mice that received intravesical PLL treatment alone was instilled, heat-denatured urine from MB49 tumor-bearing mouse bladders, and urine from MB49 tumor-bearing mouse bladders. n = 3 mice/group. (C) Volcano plot comparing the differential protein expression between MB49-bearing and control groups. Vertical lines delineate log2 (fold change) boundaries at -1 and 1; horizontal lines display the statistical significance threshold ($P < 0.05$). The blue and red dots indicate down- and upregulated genes, respectively. n = 3 mice/group. Data are presented as the means ± SEM. One-way ANOVA with Tukey's multiple comparisons test (B). ****$P < 0.0001$.
